# GLUT1 promotes cell proliferation via binds and stabilizes phosphorylated EGFR in lung adenocarcinoma

**DOI:** 10.1186/s12964-024-01678-8

**Published:** 2024-06-03

**Authors:** Zhiqing Zhou, Yu Li, Sijie Chen, Zhangrong Xie, Yuhui Du, Yue Liu, Yuxuan Shi, Xiangyi Lin, Xiaofei Zeng, Huijie Zhao, Guoan Chen

**Affiliations:** 1 Department of Human Cell Biology and Genetics, Joint Laboratory of Guangdong-Hong Kong Universities for Vascular Homeostasis and Diseases, School of Medicine, Southern University of Science and Technology, Shenzhen, 518055 China; 2 National Key Laboratory for Tropical Crop Breeding, Shenzhen Branch, Guangdong Laboratory for Lingnan Modern Agriculture, Genome Analysis Laboratory of the Ministry of Agriculture, Agricultural Genomics Institute at Shenzhen, Chinese Academy of Agricultural Sciences, Shenzhen, Guangdong 518120 China; 3 Department of Oncology, Sun Yat-sen Memorial Hospital, Sun Yat-sen University, Guangzhou, China; 4 The First Affiliated Hospital of Southern University of Science and Technology, Shenzhen, China

**Keywords:** LUAD, GLUT1, EGFR, Gefitinib, WZB117

## Abstract

**Background:**

While previous studies have primarily focused on Glucose transporter type 1 (GLUT1) related glucose metabolism signaling, we aim to discover if GLUT1 promotes tumor progression through a non-metabolic pathway.

**Methods:**

The RNA-seq and microarray data were comprehensively analyzed to evaluate the significance of GLUT1 expression in lung adenocarcinoma (LUAD). The cell proliferation, colony formation, invasion, and migration were used to test GLUT1 ‘s oncogenic function. Co-immunoprecipitation and mass spectrum (MS) were used to uncover potential GLUT1 interacting proteins. RNA-seq, DIA-MS, western blot, and qRT-PCR to probe the change of gene and cell signaling pathways.

**Results:**

We found that GLUT1 is highly expressed in LUAD, and higher expression is related to poor patient survival. GLUT1 knockdown caused a decrease in cell proliferation, colony formation, migration, invasion, and induced apoptosis in LUAD cells. Mechanistically, GLUT1 directly interacted with phosphor-epidermal growth factor receptor (p-EGFR) and prevented EGFR protein degradation via ubiquitin-mediated proteolysis. The GLUT1 inhibitor WZB117 can increase the sensitivity of LUAD cells to EGFR-tyrosine kinase inhibitors (TKIs) Gefitinib.

**Conclusions:**

GLUT1 expression is higher in LUAD and plays an oncogenic role in lung cancer progression. Combining GLUT1 inhibitors and EGFR-TKIs could be a potential therapeutic option for LUAD treatment.

**Supplementary Information:**

The online version contains supplementary material available at 10.1186/s12964-024-01678-8.

## Background

Lung cancer poses a serious public health challenge, and accounts for the highest mortality and the second-highest incidence of cancer worldwide [1]. The metabolic pattern of lung cancer is unique, shaped by complex factors such as long-term exposure to oxidative damage in lung tissues and tumor hypoxia. Lung adenocarcinoma (LUAD) represents the major subtype of lung cancer, characterized by hypoxic-induced high glucose uptake and high glucose transporter type 1 (GLUT1) expression, making the measurement of 2-deoxy-2-[fluorine-18] fluoro-D-glucose (18 F-FDG) a promising diagnostic tool [2].

GLUT1, also called SLC2A1 (Solute carrier family 2 member 1), a transmembrane protein, imports glucose from the extracellular space into the cytoplasm. GLUT1 gene overexpression has been observed in several types of cancer [3–8], including lung cancer [9, 10]. Due to its role in promoting high glucose uptake in tumor cells [11], GLUT1 is considered a potential therapeutic target for non-small cell lung cancer (NSCLC) [12, 13]. As the primary glucose transporter [14], GLUT1 supports glycolysis and mediates a complex series of tumor metabolism reprogramming events [15, 16]. Several studies have suggested that GLUT1 may contribute to epidermal growth factor receptor tyrosine kinase inhibitor (EGFR-TKI) resistance by mediating glucose metabolism [17–19]. Clinical studies have reported a correlation between EGFR expression [20] and mutation status [21] with GLUT1 expression. However, the precise mechanism through which GLUT1 regulates tumor-related signaling pathways and expression of oncogenes such as EGFR remains unclear.

EGFR, the first oncogene discovered, is a transmembrane protein and has become an eye-catching therapeutic target in lung cancer [22]. EGFR is a typical RTK, its activation usually depends on the dimerization induced by the binding of ligands to the extracellular domain, following the intracellular activation of ATP kinase site and phosphorylation of its C-terminal auto-phosphorylation domain [23]. By activating EGFR and coupling with intercellular signaling proteins, numerous tumor-related signaling pathways, such as RAS, AKT, and STAT3, are involved in tumor progression [24]. Both EGFR overexpression [25] and mutation [26] have clinical significance. Overexpression of EGFR enhances its tyrosine kinase activity by increasing the rates of EGF ligand binding with EGFR. EGFR can also be activated when mutated, which leads to a conformational change in the EGFR protein followed by dimerization and self-phosphorylation [27]. EGFR mutation not only means that it does not rely on binding to EGF ligand, but it also increases drug resistance, such as T790M, the gatekeeper mutation. Therefore, inhibiting EGFR expression in EGFR-mutated tumors has clinical benefits that can help combat drug resistance and reduce tumor growth.

Previous studies have suggested that GLUT1 may contribute to EGFR-TKI resistance by mediating glucose metabolism [17], however, the glucose metabolic independent pathway is rarely reported. Here, we report that GLUT1 is overexpressed in lung cancer and its higher expression is related to poor patient survival. GLUT1 knockdown impairs the ability of colony formation, proliferation, migration, and invasion, as well as induces apoptosis in LUAD cell lines. Mechanistically, GLUT1 is directly bonded to phosphor-EGFR and regulates phosphor-EGFR protein level via stabilizing it from ubiquitin-mediated proteolysis. Clinically, patients having higher levels of GLUT1 together with EGFR mutation have poor survival in LUAD. The use of GLUT1 inhibitor WZB117 could scientize EGFR-TKI Gefitinib in LUAD cells, suggesting that strategies aimed at the GLUT1-EGFR axis could potentially serve as an effective therapeutic approach for LUAD.

## Materials and methods

### Cell culture

The human non-small cell lung cancer (NSCLC) cell lines, including H838, H1299, H1650, H1975, PC9, and A549, were purchased from Guangzhou Cellcook Biotech Co., Ltd. These cell lines were cultured in RPMI 1640 medium (C22400500BT, Gibco, Waltham, MA, USA) supplemented with 10% fetal bovine serum (10270-106, Gibco) at 37℃ with 5% CO_2_.

### Small interfering RNA (siRNA) transfection

The human cancer cell lines were transfected with siRNAs targeting GLUT1 at a concentration of 10 µM. The transfection was performed according to the protocol of Lipofectamine RNAiMAX Reagent (13778-150, Invitrogen, Waltham, MA, USA). A negative control (NC) siRNA is also used. The siRNA sequences are listed in Supplementary Table S1.

### Drugs and reagents

MG-132 (HY-13,259, MCE, NY, USA) and CHX (HY-12,320, MCE) were dissolved by DMSO (D2650-100, Sigma, Darmstadt, Germany) to create a stock solution of 100 mM. Gefitinib (HY-50,895 MCE, NY, USA) was in a stock solution of 100µM, ensuring that the work solution contains less than 0.1% DMSO. WZB117 (HY-19,331 MCE, NY, USA) was in a stock solution of 100mM, ensuring that the work solution contains less than 0.1% DMSO. EGF (AF-100-15 Peprotech, NY, USA) was dissolved by the solvent provided by this product to create a stock solution of 1 × 10^4^Unit/µl. The critical commercial assays are listed in Supplementary Table S1.

### Colony formation assay

Human NSCLC cells were planted in 800–1500 cells per well in 6-well plates. After 8–15 days of culture, the cells were fixed with methanol and stained with crystal violet (C0121, Beyotime, Shanghai, China). The stained cells were then scanned using a scanner (Epson, Perfection v370 photo).

### Annexin V/PI flow cytometry assay

According to the protocol of Annexin V/PI kits (BMS500FI-100, Invitrogen), 1 × 10^6^ cells are stained in each sample. In the resulting image, early apoptotic cells are labeled by FITC, late apoptotic cells are labeled by both FITC and PI, and death cells are labeled by PI only.

### Cell proliferation assay

Cell proliferation was measured by Cell Counting Kit-8 (CCK-8, Yeasen, 40203ES92). Cells were planted at a density of 1000 cells per well in 96-well plates. After treatment, 10 µL CCK-8 solution was added and the cells were incubated for 1–4 h according to the protocol (Yeasen, 40203ES92). The absorbance of the wells was then read for subsequent statistical analysis.

### Transwell migration and invasion assay

Transwell of Falcone Permeable Supports (353,097, Corning, NY, USA) were used for measuring cell migration and invasion ability. The experiment of invasion was started with a layer of Matrigel (356,234, Biocoat, Corning, NY, USA). Cell suspensions with 5 × 10^4^ cells were added and fixed with methanol after 24 h. The cells were then stained with crystal violet and photographed at five randomly selected sites for subsequent statistical analysis.

### Co-immunoprecipitation (Co-IP)

The targeted proteins in the samples were combined with the corresponding antibodies after incubation. Dynabeads Protein G (10004D, Invitrogen) was used to isolate the target proteins and proteins that interact with each other in cells. Products from this experiment were used for subsequent Western Blot assay or mass spectrum analysis.

### Quantitative reverse transcription polymerase chain reaction (qRT-PCR)

The mRNA samples were obtained from cell lysis with TRIzol (15,596,026, Invitrogen, Waltham, MA, USA). RNA was purified and reverse transcribed by PrimeScript RT reagent Kit (RR047A, Takara, Kyoto, Japan) then performed real-time PCR by TB Green Premix Ex Taq II kit (RR820A, Takara, Kyoto, Japan) in Applied Biosystems 7500 (ThermoFisher, Waltham, MA, USA). The primer sequences are listed in Supplementary Table S1.

### Western blot

The protein samples were obtained from cell lysis with Cell Lysis Buffer (9803s, CST, Danvers, MA, USA) and phosphatase inhibitors (HY-K0021 and HY-K0022, MCE, NY, USA). The samples were separated using the SDS-PAGE system (L00794 and L00747, GenScript, Nanjing, China) and transferred to PVDF membranes (03010040001, Roche, Darmstadt, Germany). The primary antibodies used were PARP (9542s, CST), EGFR (4267s, CST), p-EGFR (Tyr1068) (2234, CST), GLUT1 (12939s, CST), and beta-actin (5125s, CST). The samples were incubated with primary antibodies overnight, followed by incubation with secondary antibodies anti-rabbit IgG (7074s, CST) or anti-mouse IgG (7076s, CST). The detailed information of antibodies is listed in Supplementary Table S1.

### Genomic analysis of cancer patient samples from published microarray and RNA-Seq databases

Clinical prognostic data and gene expression RNA-seq were downloaded from the TCGA cohort [28, 29], the Hou cohort [30] and the Shedden cohort [31] available on NCBI. The CEL files of microarray data were normalized using the Robust Multi-array Average (RMA) method [32]. Kaplan-Meier analysis was used to assess the probability of survival outcomes in patients with lung cancer. The log-rank test was used to obtain statistical significance between those groups.

### Tissue microarray (TMA) and immunohistochemistry (IHC)

We purchased a lung cancer tissue microarray having 98 LUAD tissues and 82 paired normal lung tissues from Shanghai Outdo Biotech Company (HLugA180Su08, Shanghai Outdo Biotech, China). All patients were pathologically diagnosed with LUAD. The TMA used in this study was approved by the Ethics Committee. IHC was performed with GLUT1 antibody (1:100, 12,939, CST, Danvers, MA). The TMA in 10 mM citrate buffer (pH 6.0) was put on medium heat in the microwave for 5 min 30 s. After treatment with 3% H_2_O_2_ for 30 min, the TMA was blocked with 10% normal goat serum and incubated with the anti-GLUT1 antibody at 4℃ overnight. The TMA was then incubated with a secondary antibody (PV-9000, ZSGB-BIO, Beijing, China) at room temperature for 1 h, stained with DAB and hematoxylin, and mounted. The slide was scanned using the Aperio VERSA - Fluorescent, Brightfield & FISH Slide Scanner. The intensity of tissues was scored from 0 to 3, representing negative staining, weak staining, moderate staining, and strong staining, respectively.

### RNA-seq

NSCLC cells were prepared for RNA-seq detection. The RNA libraries were sequenced on the illumine sequencing platform by Genedenovo Biotechnology Co., Ltd (Guangzhou, China). We analyzed the gene expression from RNA-seq using HISAT-StringTie [33] methods and performed differential expression analysis for paired-samples with DEseq2 [34].

### Data-independent acquisition mass spectrometry (DIA-MS)

For DIA-MS, the peptides were isolated and salts were removed from the protein samples. These samples were then analyzed using High-pH Reversed Phase Liquid Chromatography with the UltiMate 3000 HPLC and nano-UHPLC-MS/MS systems. The qualitative and quantitative analysis of the protein was conducted by Genedenovo Biotechnology Co., Ltd, located in Guangzhou, China.

### Statistical analysis

R V.4.2.0 (http://www.r-project.org) was utilized to perform all statistical analyses. Continuous variables were represented as the mean ± standard deviation (SD). The Student’s t-test or Wilcoxon test was used to test the differences between the two variables. Statistical significance was established at a two-tailed p-value of less than 0.05. Kyoto Encyclopedia of Genes and Genomes (KEGG) and Gene Set Enrichment Analysis (GSEA). The differentially expressed genes were analyzed by cluster Profiler [35], an R package that is commonly used to compare biological themes among gene clusters. KEGG enrichment analysis reflects the enrichment of differentially expressed genes in the KEGG pathway compared to random probability. GSEA shows the changes in KEGG pathways activation based on the differential expressed genes. Gene Ontology enrichment analysis was performed based on significantly regulated genes using the DAVID bioinformatics website [36].

## Results

### GLUT1 expression is higher in lung tumors and higher expression is related to poor patient survival in lung adenocarcinoma

GLUT1 is the most concerned glucose transporter in tumors for meeting the high glucose demand from aerobic glycolysis, which is known as the Warburg effect. It is reported that tumor hypoxia induces overexpression of GLUT1, which sustains this metabolic switch both in hypoxic and aerobic conditions [37] and promotes tumor progression. Among the genes in the glycolysis pathway, GLUT1 has the highest mRNA expression ratio of tumor/normal in TCGA LUAD cohorts measured by RNA-seq [28, 29] (Fig. S1A, Supplementary information).

**Fig. 1:**
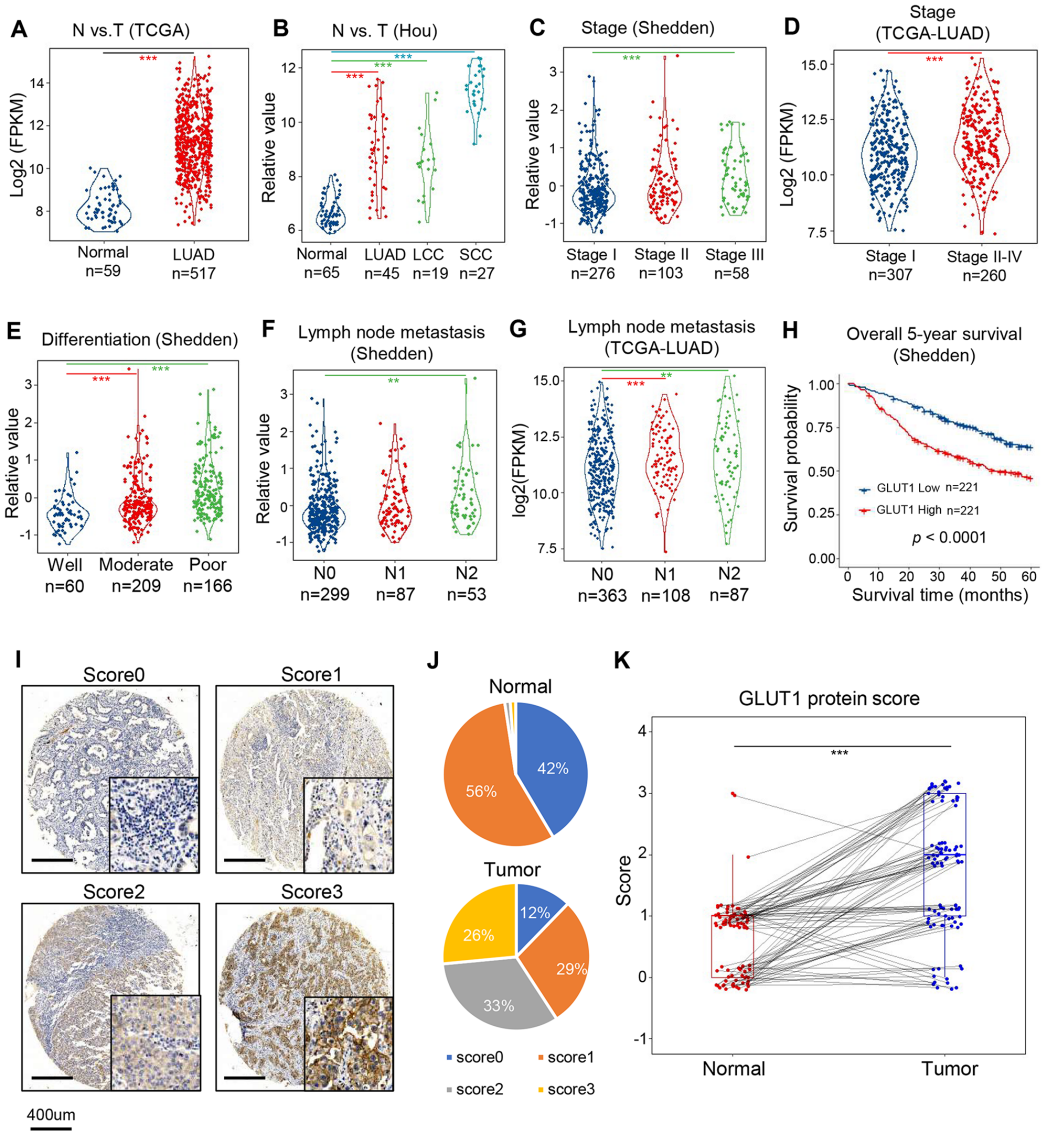
GLUT1 expression is higher in lung tumors and related to poor patient survival in lung cancer. **A**, GLUT1 mRNA expression is higher in lung adenocarcinoma (LUAD) tissues as compared to normal lung tissues in TCGA RNA-seq cohort; **B**, GLUT1 mRNA expression is higher in tumor tissue compared to normal tissues in Hou microarray cohort, LCC (large cell lung cancer), SCC (squamous cell lung cancer); **C** and **D**, GLUT1 mRNA expression is higher in later clinical stages of LUAD in Shedden microarray cohort and TCGA RNA-seq cohort; **E**, GLUT1 mRNA expression is higher in poorly differentiated LUAD in Shedden cohort; **F** and **G**, GLUT1 mRNA expression is higher in lymph node metastatic LUAD in Shedden cohort and TCGA cohort. Two-sided Wilcoxon test, ** indicates p-value < 0.01, *** indicates p-value < 0.001; **H**, Kaplan-Meier survival curve with log-rank test shows that high GLUT1 expression is related to poor 5-year survival in patients with LUAD in Shedden cohort; **I**, The represented images of TMA scored 0–3 based on their IHC staining intensity using anti-GLUT1 antibody; **J**, Percentage distribution of scores in normal tissues or tumor tissues; **K**, Paired tumor-normal samples analysis shows that GLUT1 protein is significantly highly expressed in tumors

To explore the clinical significance of GLUT1 expression, we analyzed the transcriptomic data of lung cancer from the TCGA RNA-seq cohort [28], Hou microarray cohort [30] and Shedden microarray cohort [31]. GLUT1 mRNA level is higher in LUAD, large cell (LLC), and squamous cell lung cancer (SCC) (Fig. 1A, B). Furthermore, GLUT1 is highly expressed in later clinical stages, poorly differentiated tumors, and lymph node metastasis-positive tumors (Fig. 1C-G). Moreover, high GLUT1 mRNA expression is associated with a poor five-year survival rate in patients with LUAD (Fig. 1H). Based on TCGA RNA-seq data, GLUT1 mRNAs are also highly expressed in several other types of tumors (Fig. S1B, Supplementary information).

To detect GLUT1 protein expression levels in clinical tissue specimens, we assessed it in tissue microarray including 98 LUAD (Fig. S1C, Supplementary) by IHC and scored it according to dye intensity (Fig. 1I). GLUT1 protein was predominantly found in the cell membrane. Compared to normal lung tissues, tumor tissues have a higher proportion of high scores (Fig. 1J). In 82 paired tissues, scores were higher in tumor tissues than in normal tissues (Fig. 1K).

In summary, both mRNA and protein of GLUT1 are highly expressed in lung tumor tissue compared to normal lung tissue across several public mRNA datasets and our IHC of GLUT1 protein staining. Higher GLUT1 expression may be used as a marker for diagnosis or prognosis in lung cancer.

### GLUT1 knockdown impairs colony formation and cell proliferation, and induces apoptosis of LUAD cells

**Fig. 2:**
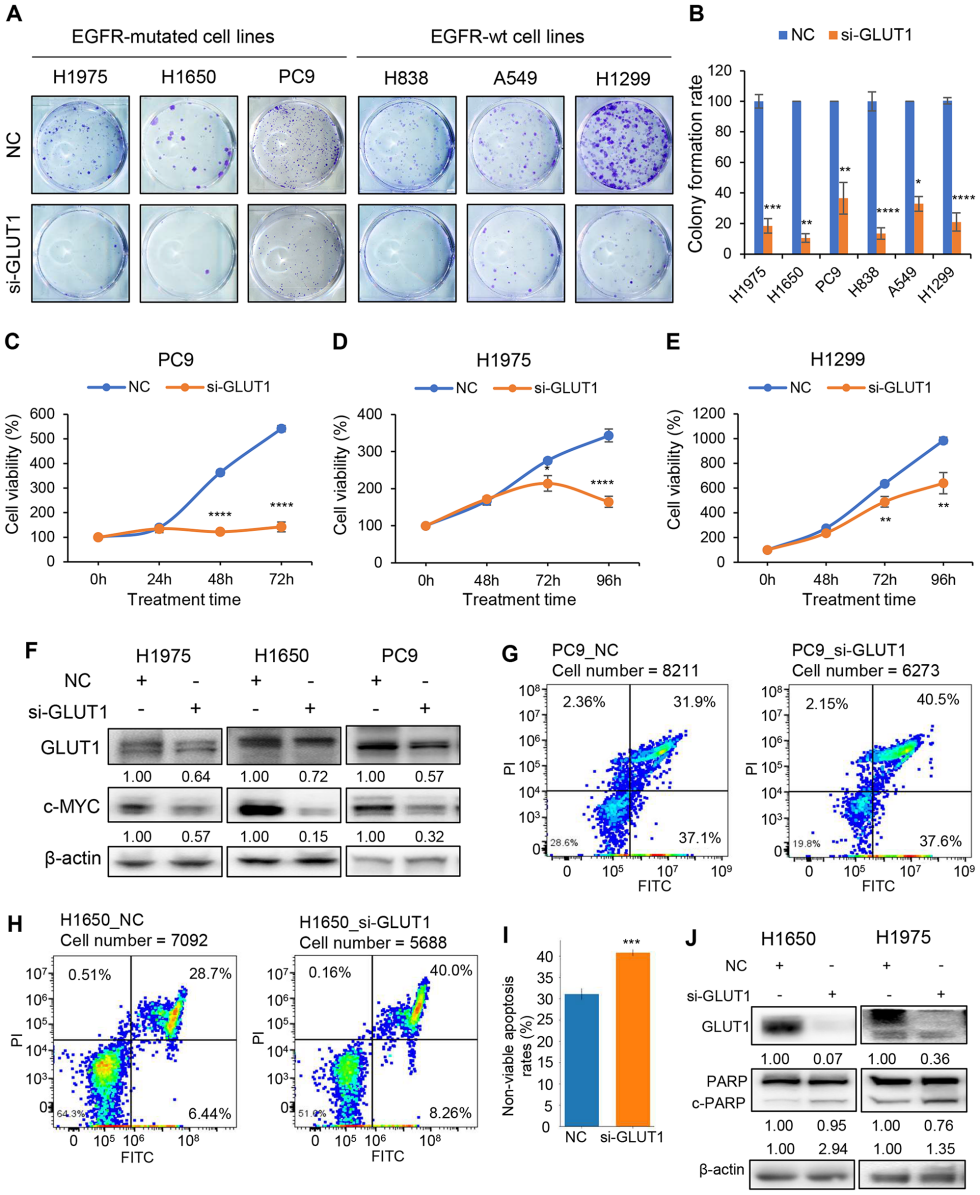
GLUT1 knockdown impairs colony formation and cell proliferation, and induces apoptosis in lung cancer cells. **A**, **B**, Colony formation ability was significantly decreased after GLUT1 knockdown in 6 NSCLC cell lines, **B** is the quantitative value of A; **C-E**, Cell proliferation was decreased after knockdown of GLUT1 for 24 h, 48 h, and 72 h in PC9, H1299, and H1975 cells. Student’s t-test, ** indicates p-value < 0.01, **** indicates p-value < 0.0001; **F**, c-MYC protein was decreased after GLUT1 knockdown in H1975, H1650, and PC9 cells; **G-I**, The percentages of apoptosis cells were increased after GLUT1 silencing in PC9 and H1650 cells; **I**, Non-viable apoptosis (late apoptosis) rate was significantly increased in GLUT1 knockdown cells; **J**, Cleaved-PARP protein level was increased after knockdown GLUT1 in H1650 and H1975 cells

To investigate the potential oncogenic roles of GLUT1, we conducted a series of cell functional experiments on lung cancer cell lines. We successfully knocked down GLUT1 using three siRNAs with different sequences in lung cancer cell lines measured by Western blotting, qRT-PCR, and RNA-seq (Figs. S2A-D, Supplementary information). For subsequent transfection assays, we combined these three siRNAs. We observed a decrease in colony formation ability and cell proliferation upon GLUT1 knockdown (Fig. 2A-E), accompanied by a decrease in the proliferation marker c-MYC protein (Fig. 2F), and an increase in the cell cycle inhibitor protein p27 (Fig. S2E, Supplementary information). This indicates that GLUT1 could affect cell growth. Next, we used flow cytometry to measure cell apoptosis and found that GLUT1 silencing induced cell apoptosis (Fig. 2G, H, and S1F). Specifically, the non-viable apoptosis (late apoptosis) rate was increased (Fig. 2I). The level of cleaved-PARP, an apoptosis marker, was also increased after the knockdown of GLUT1 (Fig. 2J), suggesting that GLUT1 could affect cell apoptosis. Taken together, GLUT1 demonstrates its oncogenic roles in cancer progression including colony formation, cell proliferation, and anti-apoptosis.

### GLUT1 silencing inhibits cell migration and invasion in LUAD cells

**Fig. 3:**
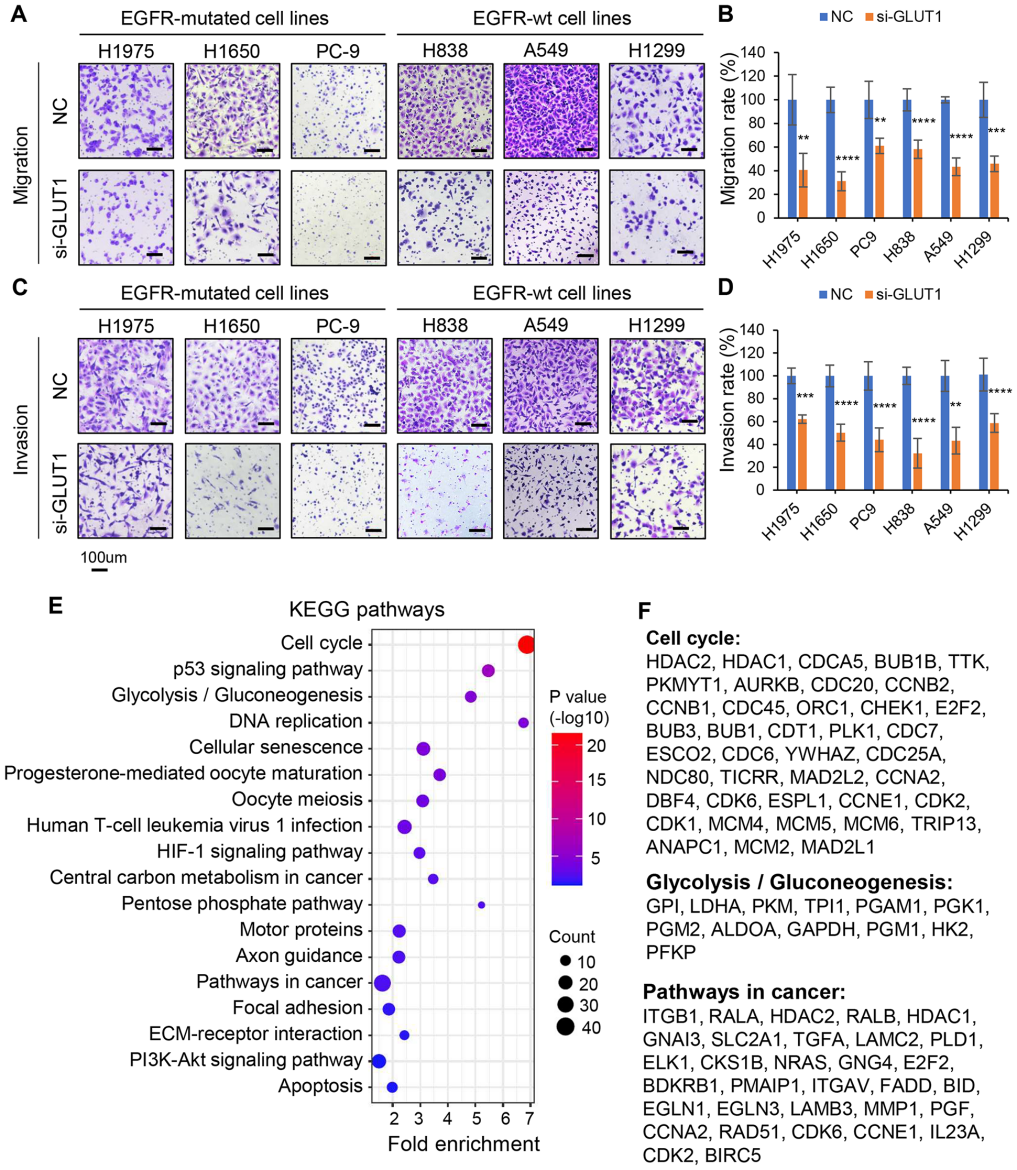
GLUT1 silencing inhibits migration and invasion in LUAD cells. **A-D**, The cell migration and invasion ability were decreased after GLUT1 silencing using siRNAs in 6 LUAD cell lines. The statistical results of the migration rate and invasion rate are shown in bar charts (**B**, **D**). Student’s t-test, *** *p* < 0.001, **** *p* < 0.0001; **E**, KEGG pathway analysis using these 686 positively correlated with GLUT1 genes showing that GLUT1 was involved in cell cycle and several cancer-related signaling pathways; **F**, Gene list of Cell cycle, Glycolysis/Gluconeogenesis and Pathways in cancer

Cancer metastasis is the leading cause of death in patients with lung cancer. Cancer cells have higher migration and invasion ability and more metastasis potential [38]. To investigate whether GLUT1 could affect cell migration and invasion in vitro. We perform Transwell assays to detect cell migration and invasion ability after GLUT1 knockdown in lung cancer cell lines. Upon GLUT1 silencing, the cell migration and invasion ability were significantly decreased in 6 tested NSCLC cell lines (Fig. 3A-D), suggesting GLUT1 may have a role in promoting cancer metastasis.

To explore the role of GLUT1 in human LUAD tissues, we download three RNA-seq datasets from public lung cancer datasets, the UM cohort (67 LUAD) [29], the SEO cohort (85 LUAD) [39] and the TCGA cohort (312 LUAD) [28, 29]. Next, we performed the Pearson correlation analysis between GLUT1 mRNA and all other genes in these 3 LUAD datasets. There are 686 genes positively correlated with GLUT1 mRNA expression in LUAD tissues (Pearson correlation, *n* = 464, average *r* > 0.34, *p* < 0.01). Using these 686 genes, we performed KEGG analysis on the DAVID website. We found that the GLUT1 positively correlated genes were involved in the cell cycle and several cancer-related signaling pathways (Fig. 3E, F), including Cell cycle, p53 signaling pathway, Glycolysis/Gluconeogenesis, DNA replication, Cellular senescence, HIF-1, Pathways in cancer, Focal adhesion, PI3K-AKT signaling pathway, and Apoptosis. GO BP (Gene Ontology, Biology Process) analysis of these 686 genes shows that the cell cycle is also on the top list, which further supports that GLUT1 knockdown impaired cell proliferation in vitro shown in Fig. 2C-E. Taken together, GLUT1 is involved not only in the Glycolysis/Gluconeogenesis pathway but also in cell cycle and cancer-related pathways, which enhance cell proliferation, migration, invasion, and metastatic capabilities in lung cancer progression.

### GLUT1 protein physically interacts with phosphor-EGFR protein and regulates EGFR singling pathway

**Fig. 4:**
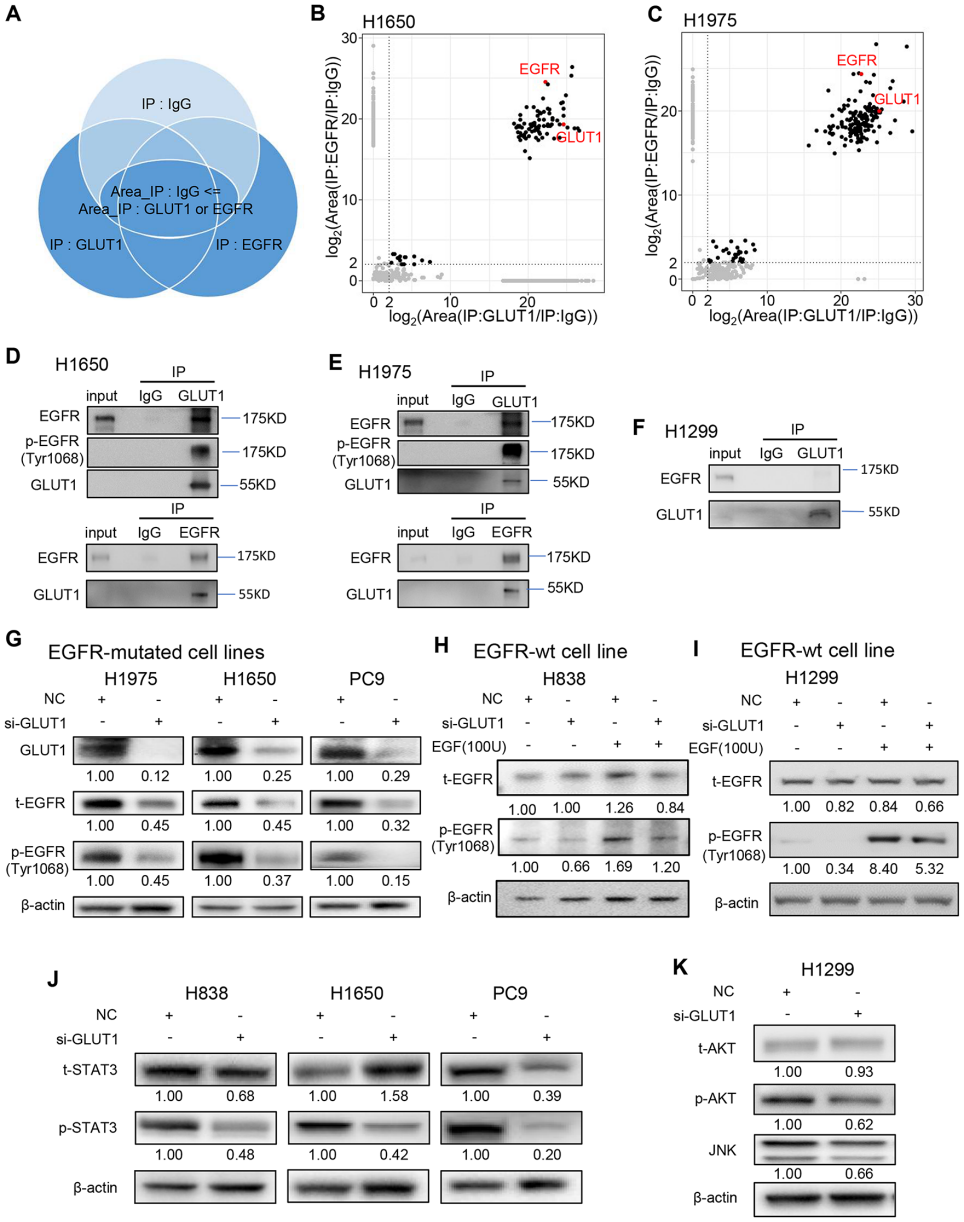
GLUT1 protein physically interacts with phosphorylated EGFR protein and regulates EGFR singling pathway. **A**-**C**, LCMS (Liquid chromatography-mass spectrometry) was used to identify the Co-IP binding proteins. IgG, EGFR, and GLUT1 antibodies were used to pull down proteins in H1650 and H1975 cell lines. Proteins pull-down by IgG as negative control. **A**, Proteins interacting with EGFR or GLUT1 are selected based on two screening criteria: protein pull-down by IgG is negative or protein area detected by IgG is less than 2 folds by EGFR or GLUT1; **B, C**, The selected proteins are shown, EGFR and GLUT1 are labeled in red; **D**-**F**, Western Blotting was used to verify the protein Co-IP results. GLUT1 was detected in protein samples that pull down by EGFR antibody, and EGFR has also been detected in protein samples that pull down by GLUT1 antibody both in H1650 and H1975, but not in H1299; **G**, EGFR protein levels were decreased upon GLUT1 silencing in EGFR-mutated cell lines, H1975, H1650 and PC9; **H, I**, Only the p-EGFR protein was decreased after GLUT1 silencing in EGFR-wt cell line H838 and H1299; **J, K**, EGFR downstream proteins, p-STAT3, p-AKT, and JNK were decreased upon GLUT1 silencing

To identify potential proteins that might bind to GLUT1, we performed Co-IP-MS with GLUT1 antibody in H1975 and H1650 cell lines, using IgG antibody as the negative control. Protein level was represented by area, and the fold change of GLUT1 area/IgG area large 2 was selected as potential binding proteins. We found that the EGFR protein was one of the identified proteins pulled down by GLUT1 antibody in both H1650 and H1975 cells (Fig. 4A-C, and Fig. S4A, B, Supplementary information). EGFR is one of the key oncogenes in NSCLC. It forms dimer and phosphorylates when it has mutations or interacts with EGF ligands. To confirm if GLUT1 was bound to EGFR protein, we performed Co-IP-MS with EGFR antibody in H1975 and H1650 cell lines and found that GLUT1 was identified on the EGFR protein binding list (Fig. 4A-C, and Fig. S4C, D, Supplementary information). Proteins identified in all four experimental groups in terms of detection (fold change to IgG large 2) were listed in Supplementary Table S2.

Next, we performed Co-IP-Western blotting to verify the direct binding of GLUT1 and EGFR protein. GLUT1 binding with EGFR was confirmed in H1650 and H1975 cells (Fig. 4D, E). As we know H1650 and H1975 cells are both EGFR mutated cells, which predominantly have auto-phosphor-EGFR protein only. The antibody used in our Co-IP experiment is an anti-total-EGFR antibody, which can interact with both the total-EGFR and phosphor-EGFR (p-EGFR) proteins. So, we perspective GLUT1 may interact with p-EGFR protein only. As expected, a binding band was detected when we used an anti-p-EGFR (Tyr1068) antibody in the GLUT1 pull-down assay in both H1650 and H1975 cell lines (Fig. 4D, E). H1299 is an EGFR-wild type cell line, which has little p-EGFR protein if without EGF ligand stimulation. We found that there was no or weak GLUT1-EGFR binding band when using an anti-total-EGFR antibody in H1299 (Fig. 4F). However, a stronger EGFR binding band was detected upon EGF stimulated in H1299 (Fig. S4G, Supplementary information), further indicating that GLUT1 may interact with p-EGFR protein only.

Several studies have pointed out that GLUT1 expression is significantly positively correlated with EGFR expression or EGFR mutation status [40, 41], however, the potential mechanism is still unknown. To further explore if GLUT1 affects EGFR expression, we measured EGFR expression levels in both mRNA and protein, as well as proteins in its singling pathway. We found that the EGFR mRNA expression was not changed after GLUT1 knockdown (Fig. S4E, F, Supplementary information), indicating that the regulation of GLUT1 on EGFR is not at the transcriptional level. Since GLUT1 may directly bind to p-EGFR, we sought to determine whether GLUT1 affects EGFR protein expression. We found that the p-EGFR protein was decreased upon GLUT1 silencing in three EGFR-mutated lung LUAD cell lines (H1975, H1650, and PC9) (Fig. 4G). The bands of the total EGFR protein are the same as p-EGFR protein indicating that the major EGFR protein is p-EGFR since the anti-total EGFR antibody can also reorganize p-EGFR protein. As we know, in EGFR-wt (wild type) cell lines (H838 and H1299), the major form of EGFR is un-phosphor-EGFR if there is no EGF stimulation. As expected, the p-EGFR protein was down-regulated after GLUT1 knockdown when EGF was stimulated in both H838 and H1299 cells (Fig. 4H, I). We didn’t find if over-GLUT1 can increase EGFR protein level (Fig. S4H, Supplementary information), suggesting GLUT1 can only prevent EGFR degradation, not promote EGFR expression.

The phosphorylated EGFR can activate downstream signaling pathways, such as AKT, ERK, and STAT3, involving cell proliferation, survival, and invasion. Recent studies have shown that EGFR is involved in metabolic reprogramming in tumors. For example, EGFR can increase glucose supply by interacting with and stabling SGLT1 (sodium/glucose cotransporter 1) but not GLUT1 [42]. Finally, we delved into the EGFR downstream signaling pathway and observed that several EGFR signaling-related proteins such as p-AKT, p-STAT3, JNK, and p-ERK were decreased upon GLUT1 knockdown (Fig. 4J, K, and S4I, Supplementary information), suggesting GLUT1 could activate EGFR signaling pathway in lung cancer.

As described above, we first uncovered that GLUT1 interacts directly with p-EGFR protein and regulates p-EGFR protein level and its downstream singling pathways, suggesting that GLUT1 has a glucose metabolic-independent role. Next, we investigated the regulation and underlying mechanism of this interaction.

### GLUT1 prevents EGFR protein degradation via ubiquitin-mediated proteolysis and regulates several oncogenic signaling pathways

**Fig. 5:**
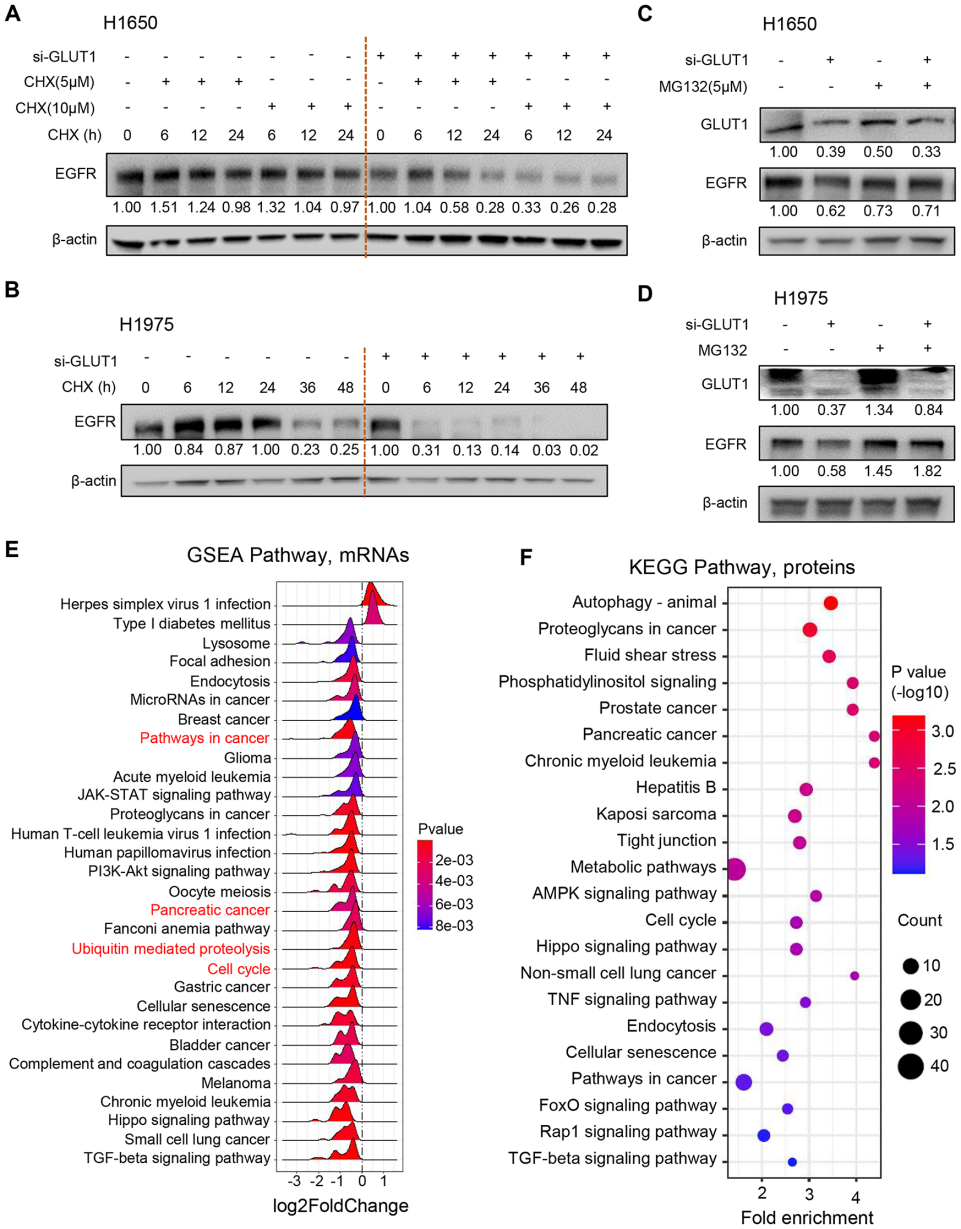
GLUT1 prevents EGFR protein degradation via ubiquitin-mediated proteolysis and regulates several oncogenic signaling pathways. **A**, **B**, EGFR protein level with GLUT1 knockdown and CHX (Cycloheximide) treatment. EGFR protein half-life is shortened with GLUT1 silencing; **C**, **D**, EGFR protein level with GLUT1 silencing and MG132 treatment. MG132 treatment can rescue partially the downregulation of EGFR after GLUT1 silencing; **E**, The GSEA (Gene Set Enrichment Analysis) pathway enrichment analysis of the RNA-seq data from three NSCLC cell lines (PC9, H1650, and H1975). Up/down-regulated (si-GLUT1/NC Control); **F**, KEGG pathway analysis of 426 down-regulated proteins (siGLUT1/NC control < 0.65 in 2/3 cell lines) in DIA-MS data from three LUAD cell lines, PC9, H1650, and H1975

We have revealed that GLUT1 can directly bind to p-EGFR and affect EGFR at the translational level. To investigate the underlying mechanism, we first measured the EGFR protein half-life using the protein synthesis inhibitor cycloheximide (CHX) in H1650 and H1975 cells. We observed a faster degradation of EGFR protein in GLUT1 knocked down cells (Fig. 5A, B), indicating GLUT1 can affect EGFR protein half-life in living cells. Next, to test whether GLUT1 affects EGFR protein stability through the ubiquitin-proteasome pathway, we treated the cells with the proteasome inhibitor MG132 and measured the changes in EGFR protein level. We found that GLUT1 knockdown reduced EGFR protein levels, but this effect was rescued partially by MG132 treatment (Fig. 5C, D). We also found that the ubiquitination lever was decreased after GLUT1 knockdown using anti-ubiquitin antibody (Fig. S5A). We concluded that GLUT1 physically binds to p-EGFR and prevents EGFR protein degradation via ubiquitin-mediated proteolysis. Further investigating the protein binding site and regulating by which E3 ligase becomes more interesting.

We previously analyzed the GLUT1-correlated genes in human LUAD tissues and found that GLUT1-correlated genes were involved in the cell cycle and cancer-related signaling pathways. To further explore the potential molecular singling pathways regulated by GLUT1 in lung cancer cell lines, we performed RNA-seq and DIA-MS analysis following GLUT1 silencing in three LUAD cell lines, H1650, H1975, and PC9. We first performed GSEA analysis to enrich signaling pathways affected by GLUT1 knockdown based on RNA-seq data. The differential expressed genes (siGLUT1/NC control) were enriched in the Cell cycle, Pathways in cancer, JAK-STAT, PI3K-AKT, TGF-beta, Hppo, Focal adhesion, etc. (Fig. 5E), which was similar to previously analyzed results based on GLUT1 correlated genes from LUAD tissues (Fig. 3E). Interestingly, ubiquitin-mediated proteolysis signaling was involved in GLUT1 regulation (Fig. 5E).

The differential expressed genes were selected based on this RNA-seq data. There are 616 downregulated genes using the critical of siGLUT1/NC control < 0.65 in 2/3 cell lines. Then, we performed KEGG pathway analysis based on these 616 downregulated genes using the DAVID website. The results of KEGG pathway analysis also indicated that Hippo, Cell cycle, Pathways in cancer, p53, TGF-beta, etc. were affected by GLUT1, which were similar to GSEA analysis (Fig. S5B, Supplementary information).

From the DIA-MS data, there were 426 downregulated proteins if siGLUT1/NC control < 0.65 in 2/3 cell lines. These 426 downregulated proteins were enriched in the pathways related to Autophagy, Metabolic, Cell cycle, Pathways in cancer, TGF-beta, etc. (Fig. 5F). From RNA-seq and DIA-MS data following GLUT1 silencing, both the transcriptional and translation levels were affected by GLUT1 suggested that GLUT1 is involved in the regulation of large genes or pathways in LUAD, such as Cell cycle, Pathways in cancer, TGF-beta and ubiquitin- ubiquitin-mediated proteolysis.

In summary, GLUT1 could bind and prevent EGFR protein degradation by undergoing ubiquitin-mediated proteolysis signaling. GLUT1 promotes cancer progression through the regulation of EGFR, cell cycle, and oncogenic signaling pathways.

### GLUT1 inhibitor WZB117 can enhance Gefitinib sensitivity to LUAD cells

**Fig. 6:**
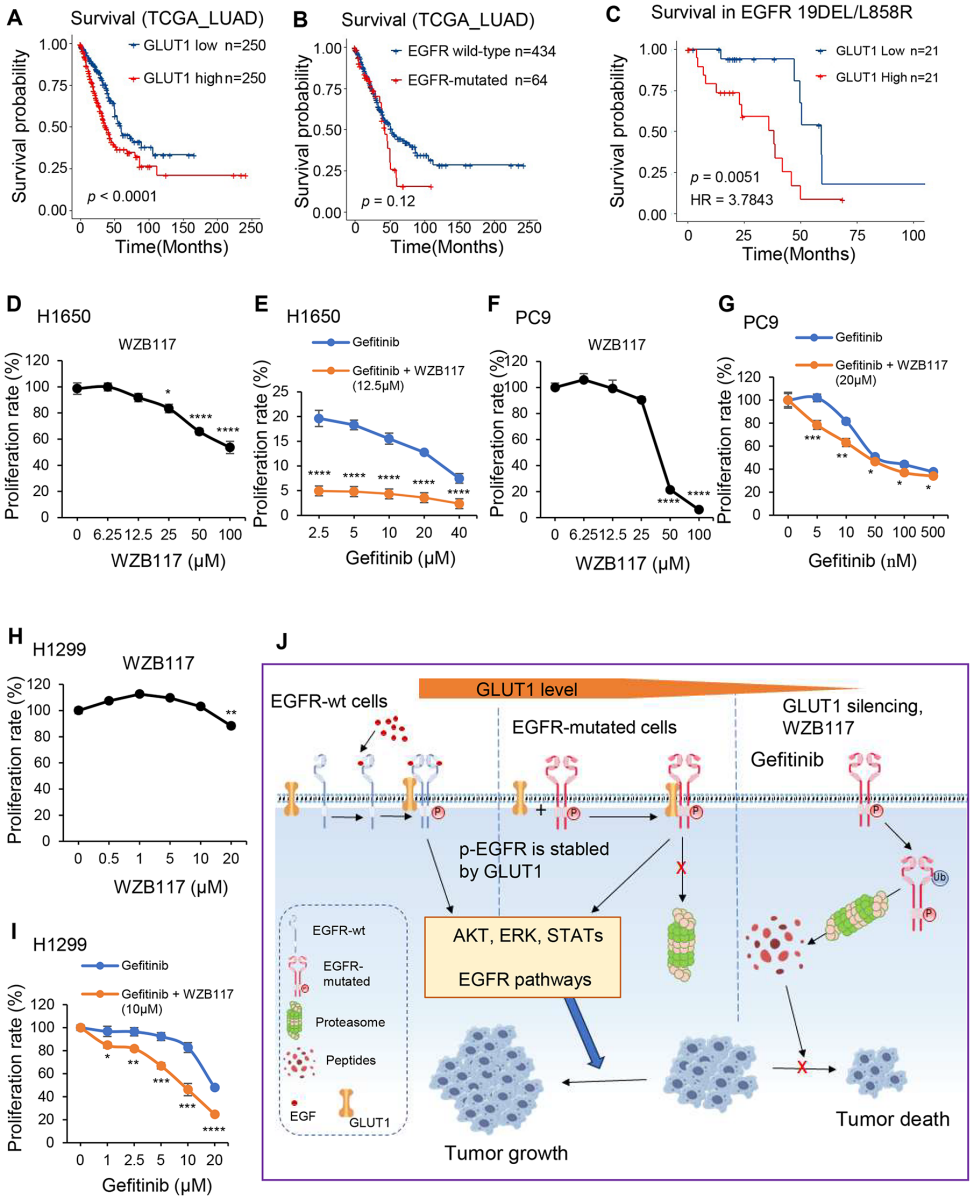
GLUT1 inhibitor WZB117 can enhance Gefitinib sensitivity to LUAD cells A-C, Kaplan-Meier survival curves showing the subgroups of samples according to the presence of EGFR mutation and GLUT1 expression in the TCGA-LUAD cohort. **D**, Cell proliferation rate after a different dose of WZB117 treatment in H1650 cell line; **E**, Cell proliferation rate after a different dose of Gefitinib or combined with 12.5µM WZB117 treatment in H1650 cell line; **F**, Cell proliferation rate after a different dose of WZB117 treatment in PC9 cell line; **G**, Cell proliferation rate after different dose of Gefitinib or combined with 20µM WZB117 treatment in PC9 cell line; **H**, Cell proliferation rate after different dose of WZB117 treatment in H1299 cell line; **I**, Cell proliferation rate after different dose of Gefitinib or combined with 10µM WZB117 treatment in H1299 cell line; **J**, Working model of GLUT1 in regulation of EGFR signaling pathway in LUAD. High levels of GLUT1 can bind and prevent EGFR protein degradation to active EGFR signaling pathways and promote tumor progression. While, a low level of GLUT1, GLUT1 inhibitor WZB117, or combined WZB117 with Gefitinib can block the EGFR signaling pathway and inhibit tumor growth

We previously found that higher GLUT1 expression was correlated with poor patient survival in the Shedden-LUAD cohort (Fig. 1H). It’s also related to unfavorable survival in TCGA-LUAD cohort (Fig. 6A). However, EGFR mutation status does not correlate with survival rate in TCGA-LUAD cohort (Fig. 6B). Further detailed analysis by combining GLUT1 mRNA level and EGFR mutation status revealed that the group of samples having higher GLUT1 expression level together with EGFR mutations showed a poor survival rate compared to other groups in TCGA-LUAD cohort (Fig. 6C).

We next test if GLUT1 inhibitor WZB117 has a role in cell growth in LUAD cells. We found that cell proliferation was decreased upon WZB117 treatment in different dosages (Fig. 6D, F, H, and S6C). Gefitinib, the first-generation EGFR-TKI, is recommended as the first-line treatment for LUAD with Del19 EGFR mutation. Then we wonder if WZB117 can enhance Gefitinib sensitivity to LUAD cells. When combined with a low concentration of WZB117, there is a significant reduction in the proliferation rate compared to using Gefitinib treatment alone in Del19 EGFR-mutated LUAD cells, H1650 and PC9 (Fig. 6D-G). Although Gefitinib is not the first-line treatment of NSCLC without EGFR mutation, we observed that drug sensitivity also increased when combined with WZB117 treatment in H1299 (Fig. 6H, I). Interestingly, we found that WZB117 enhances Gefitinib drug sensitivity in DEL19 cells (H1650 and PC9) but not in T790M and L858R EGFR mutation cell line, H1975 (Fig. S6A, B, Supplementary information). We have applied a third-generation EGFR TKI Osimertinib in the H1975 cell line and found that WZB117 also cannot enhance Osimertinib drug sensitivity in this cell line (Fig. S6C, Supplementary information).

Overall, the presence of higher GLUT1 expression along with an EGFR mutation could potentially serve as a poor prognostic marker in LUAD, although the underlying mechanism remains unclear. GLUT1 interacts directly with p-EGFR protein and activates EGFR signaling pathways to promote tumor progression. GLUT1 inhibitors could enhance the sensitivity of both DEL19 and wild-type LUAD cells to the EGFR inhibitor Gefitinib. These results suggest that targeting GLUT1 or in combination with EGFR-TKIs could potentially be used for the treatment of LUAD (Fig. 6J).

## Discussion

GLUT1 is widely recognized as a cancer biomarker and a key regulator of glucose metabolism in cancers. Disturbance in glucose metabolism is a significant characteristic of lung cancer [15], which positions GLUT1 as a potential marker for aggressive progression and poor prognosis [10]. Zhang et al. analyzed the relationships between GLUT1 protein expression and clinicopathological parameters across 26 studies. They found that high GLUT1 expression significantly predicts a poor prognosis in lung cancer [43]. In this study, we revealed that GLUT1 is overexpressed at both the transcriptional and translational levels in lung cancer. Overexpression of GLUT1 is associated with advanced clinical stages, poor differentiation, lymph node metastasis, and poor patient survival rate. Further, the presence of EGFR mutation along with higher GLUT1 expression shows an even worse survival in patients of LUAD. Our study provides additional evidence to support that GLUT1 may be used as a diagnosis and prognosis marker.

Tumor growth and metastasis are the primary causes of mortality in lung cancer patients. We found that after GLUT1 silencing, there was a decrease in cell proliferation and colony formation ability, while apoptotic cells increased. This suggests that GLUT1 can affect tumor growth ability. The ability of tumor cells to migrate and invade is a key factor in promoting metastasis. We noted a decrease in migration and invasion ability following GLUT1 knockdown. These results indicate that GLUT1 has an oncogenic role in cancer progression.

Glycolysis plays a crucial role in the reprogramming of tumor metabolism. The overexpression of GLUT1, which is often seen as the initial step in the metabolic shift of tumors, caters to the high glucose requirements necessary for tumor growth and progression. Our KEGG analysis of GLUT1 correlated genes from human LUAD tissues and RNA-seq and DIA-MS data from cell lines revealed that the expression of GLUT1 influences the cell cycle, glycolysis/gluconeogenesis, and several cancer-related signaling pathways. These pathways are known to be involved in regulating the hallmarks of cancer, such as cell proliferation, invasion activation, and resistance to cell death, thereby controlling tumor growth and progression. All these results suggested that GLUT1 is a pivotal factor in understanding the relationship between tumor metabolic reprogramming and the activation of tumor-related non-metabolic signaling pathways.

EGFR is one of the most well-characterized oncogenic drivers of LUAD [44]. The phosphorylated EGFR can activate downstream signaling pathways, including MAPK/ERK, PI3K/Akt/mTOR, and JAK/STAT pathways. These pathways play a critical role in cell proliferation, survival, invasion, and metastasis [45]. Previous studies have reported a complex mutual regulation network between EGFR and aerobic glycolysis. Both EGFR and GLUT1 are positively correlated with 18-FDG uptake and regulate glucose metabolism in NSCLC [46]. EGFR promotes glycolysis by stabilizing Sodium-glucose co-transporter 1 (SGLT1) [47]. The activation of PI3K/AKT signaling pathways, which are downstream of EGFR, may enhance glycolysis by upregulating GLUT1 expression [48, 49] or translocating GLUT1 to the plasma membrane [50, 51]. Moreover, robust glycolysis is necessary for EGFR-mutated to protect EGFR from autophagy-mediated degradation in NSCLC [52]. However, despite GLUT1 being one of the most commonly overexpressed genes related to tumor glycolysis, further exploration of the intrinsic relationship with EGFR is lacking. Here, we reported that GLUT1 can directly interact with phosphor-EGFR and prevent EGFR from ubiquitin-mediated proteolysis in LUAD cells. This study presents a new molecular mechanism in EGFR regulation by GLUT1, a glycolysis-related glycose transporter, that could bind and stabilize EGFR in LUAD.

There are several major phosphorylation sites in the EGFR auto-phosphorylation domain, such as Y992, Y1045, Y1068, Y1086, Y1148, Y1173, etc [53]. Most of these phosphorylation sites are phosphorylated upon any EGFR mutation or EGF stimulation [53]. In this study, we have chosen the EGFR Tyr1068 antibody for Western and Co-IP since the Tyr1068 site is the most used because the anti-EGFR Tyr1068 antibody is more specific, easily detected, and more sensitive to Gefitinib [54]. We have tested Tyr1086 using an anti-EGFR Tyr1086 antibody, and there is no band. We didn’t test other phosphorylation sites in this study.

Clinical studies have reported the correlations between EGFR and GLUT1. Both GLUT1 and EGFR are most frequently expressed in both primary and metastatic tumors [55]. In clinical cases of breast cancer, a significant association was found between GLUT1 expression and EGFR [56]. In our study, we found no significant difference in survival rates between the EGFR mutation group and the EGFR-wild type group in LUAD. However, when we consider it in the equivalent expression level of GLUT1, significant differences emerged between these two groups. Furthermore, GLUT1 expression levels were found to determine patient survival time in conjunction with EGFR mutation. This suggests that GLUT1 expression and EGFR mutation status might jointly determine clinical prognosis.

Gefitinib, a first-generation EGFR-TKI, is also the first-line treatment of T790M negative EGFR-mutation LUAD. It has been reported that high total lesion glycolysis is associated with a higher response rate and the development of EGFR-TKI resistance when treated with Gefitinib [57]. However, the combination of EGFR-TKI and glycolysis inhibitor has not yet been studied. Here, we found that GLUT1 inhibitor WZB117 can increase Gefitinib sensitivity in LUAD cells with EGFR Del19 mutation (H1650 and PC9). While Gefitinib is not used clinically in EGFR-wild type NSCLC, we found that WZB117 also can enhance Gefitinib sensitivity in EGFR-wild type NSCLC cells (H1299). This has been reported by Suzuki et al. in A549 cell lines [17], suggesting that the combination of GLUT1 inhibitors with Gefitinib could potentially broaden the scope of treatment in LUAD. However, WZB117 was not able to overcome the Gefitinib or Osimertinib resistance in T790M EGFR mutated LUAD (H1975). This study further revealed the molecular mechanism of how GLUT1 regulates EGFR and provides new theoretical support for considering GLUT1 as a potential target in EGFR-TKI combination therapy.

This study aims to elucidate the role and molecular mechanisms of GLUT1 in LUAD. We observed that GLUT1 physically interacts with p-EGFR protein in LUAD cell lines, however, the specific binding sites are unknown. From our Co-IP-MS results, we got several peptides of EGFR or GLUT1 proteins from H1650 and H1975 cells. There is a common peptide (2012–2022 of EGFR, extracellular domain) detected in both H1650 and H1975 (data not shown). We didn’t get peptides from the auto-phosphorylation domain (979–1210) of EGFR protein, suggesting the binding site of EGFR may be not at the auto-phosphorylation domain. The specific binding sites require further exploration. The identity of the E3 ligase that leads to EGFR proteolysis upon GLUT1 silencing is also worth investigating. Additionally, it is still to be determined whether GLUT1 and WZB117 can help reduce the acquired resistance of Gefitinib.

## Conclusion

This study discovered that GLUT1 is highly expressed in lung cancer, and overexpression is associated with unfavorable survival in patients with LUAD. Higher GLUT1 expression levels together with EGFR 19DEL or L858R mutation status have poor patient survival in LUAD. The cell proliferation, colony formation, migration, and invasion were impaired upon GLUT1 silencing. The cell apoptosis was also induced after GLUT1 knockdown. GLUT1 was involved in the cell cycle, p53 signaling pathway, glycolysis/ gluconeogenesis, and several cancer-related pathways revealed by human LUAD tissues and cell lines. GLUT1 directly interacts with p-EGFR and prevents EGFR from undergoing ubiquitin-mediated proteolysis. GLUT1 knockdown can decrease p-EGFR protein expression and its downstream AKT, ERK, and STAT3 signaling pathways. Additionally, GLUT1 inhibitor WZB117 can enhance the Gefitinib sensitivity in LUAD cells. GLUT1 could serve as a diagnostic and prognostic marker and a potential therapeutic target by combining GLUT1 inhibitor with EGFR-TKI Gefitinib in LUAD.

### Electronic supplementary material

Below is the link to the electronic supplementary material.

Supplementary Material 1

## Data Availability

The datasets supporting the conclusions of this article are included within the article.

## Abbreviations

GLUT1: Glucose transporter type 1
SLC2A1: Solute carrier family 2, member 1
SGLT1: Sodium-glucose co-transporter 1
EGFR: Epidermal growth factor receptor
NSCLC: Non-small cell lung cancer
LUAD: Lung adenocarcinoma
LLC: Large cell lung cancer
SCC: Squamous cell lung cancer
siRNA: Small interfering RNAs
WZB117: 2-fluoro-6-(m-hydroxybenzoyloxy) phenyl m-hydroxybenzoate
CHX: Chloroquine, an autophagy inhibitor
MG132: Proteasome inhibitor
GSEA: GeneSet Enrichment Analysis
TCGA: The Cancer Genome Atlas Program
18F-FDG: 2-deoxy-2-[fluorine-18] fluoro-D-glucose
TKI: Tyrosine kinase inhibitor
TMA: Tissues microarray
IHC: Immunohistochemistry
KEGG: Kyoto Encyclopedia of Genes and Genomes
ATP: Adenosine Triphosphate
RTKs: Receptor tyrosine kinases
