# Supplementary material for: GLUT1 promotes cell proliferation via binds and stabilizes phosphorylated EGFR in lung adenocarcinoma
Source: Cell Commun Signal. 2024 Jun 3;22:303. doi: 10.1186/s12964-024-01678-8 (PMC11145837; doi:10.1186/s12964-024-01678-8)
Supplement: Supplementary file 1 — Supplementary Material 1 [file 12964_2024_1678_MOESM1_ESM.pdf]

Supplementary Figure S1

A Genes in glycolysis pathway (TCGA RNA-seq)

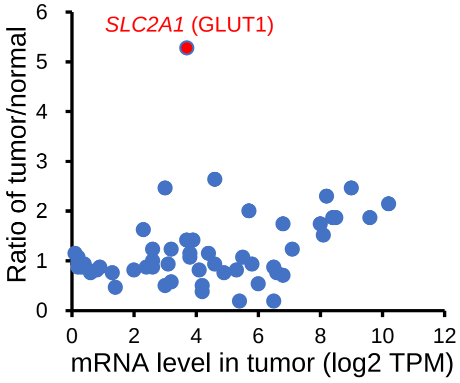

B

GLUT1 (SLC2A1) mRNA expression profile across all tumor samples and paired normal tissues

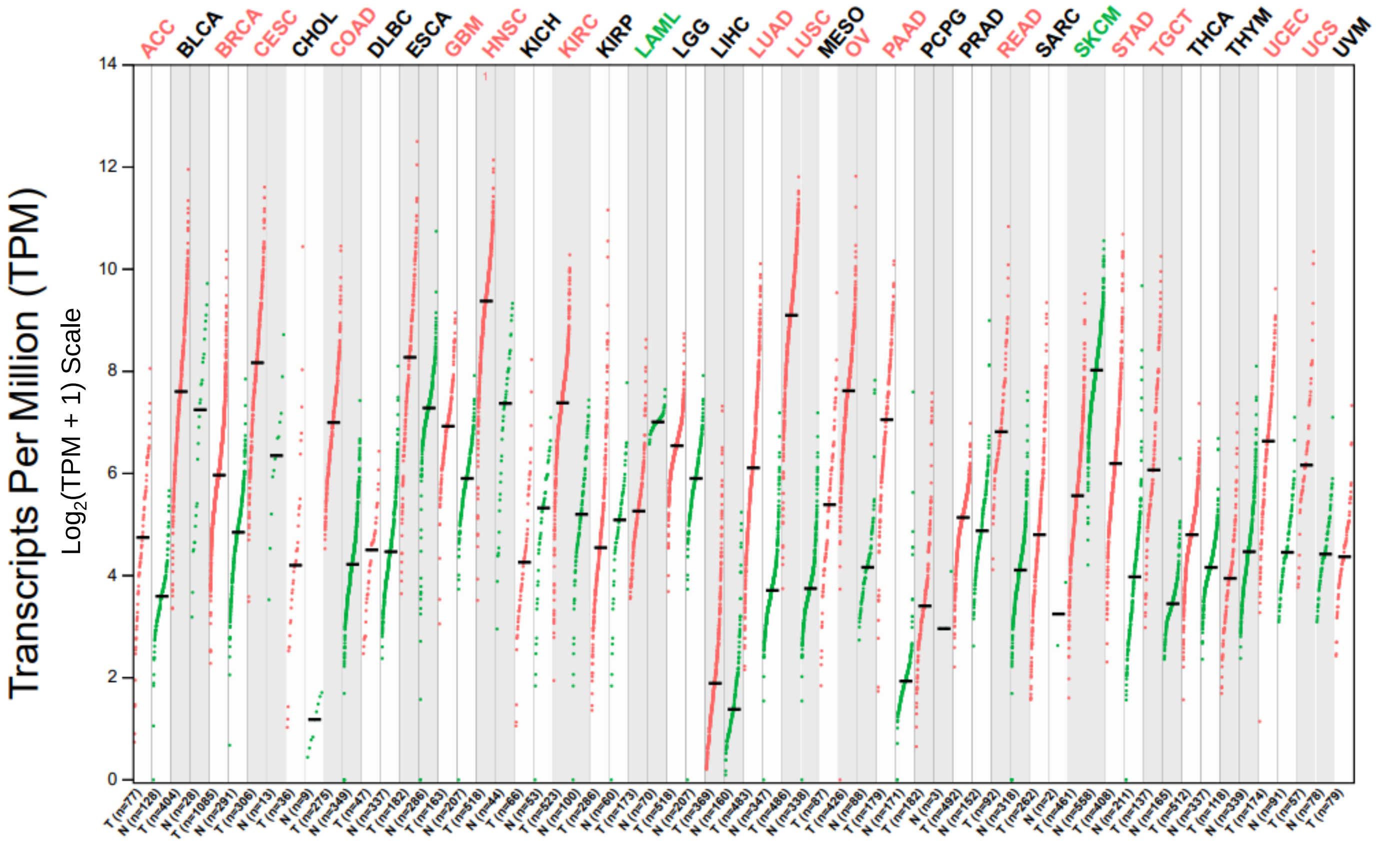

Number of samples

Each dot represents the expression of sample

Supplementary Figure S1

C Lung TMA IHC

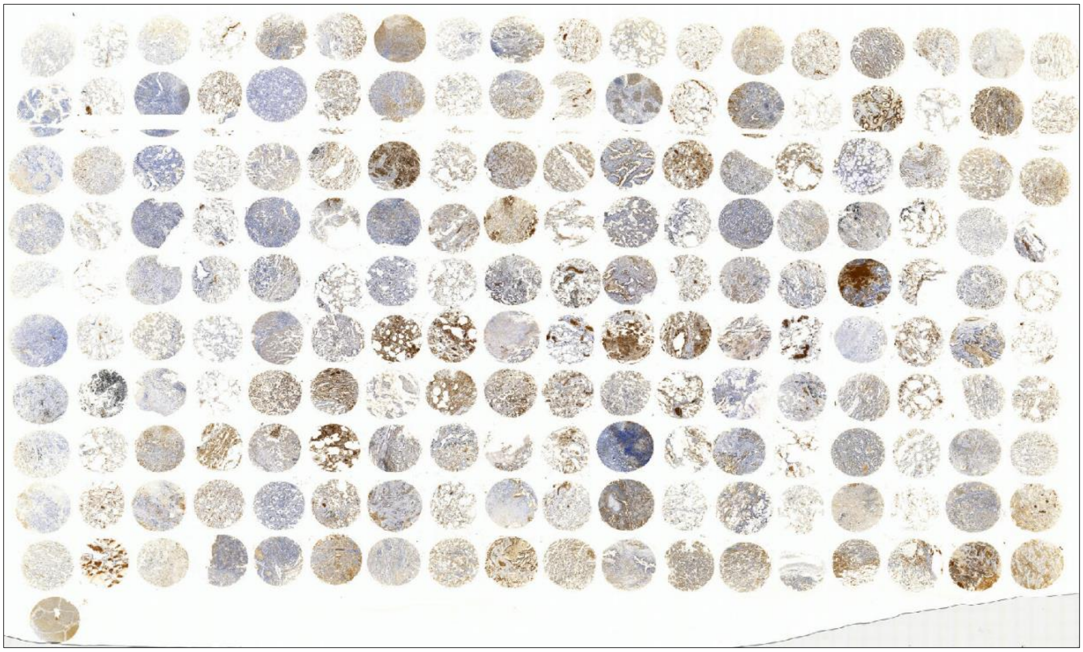

Figure S1. GLUT1 is overexpressed in lung tumors. A, GLUT1 mRNA shows the highest fold changes (tumor/normal) in glycolysis-related genes from TCGA RNA-seq data; **B**, GLUT1 mRNAs are highly expressed in multiple types of tumors in TCGA data from the GEPIA website, red or green indicating Tumor vs. Normal, $p < 0.05$; **C**, Immunohistochemistry (IHC) of tissue microarray (TMA) with paired normal and lung tumor tissues (n=82) and not paired tumor tissues (n=16) in LUAD.

Supplementary Figure S2

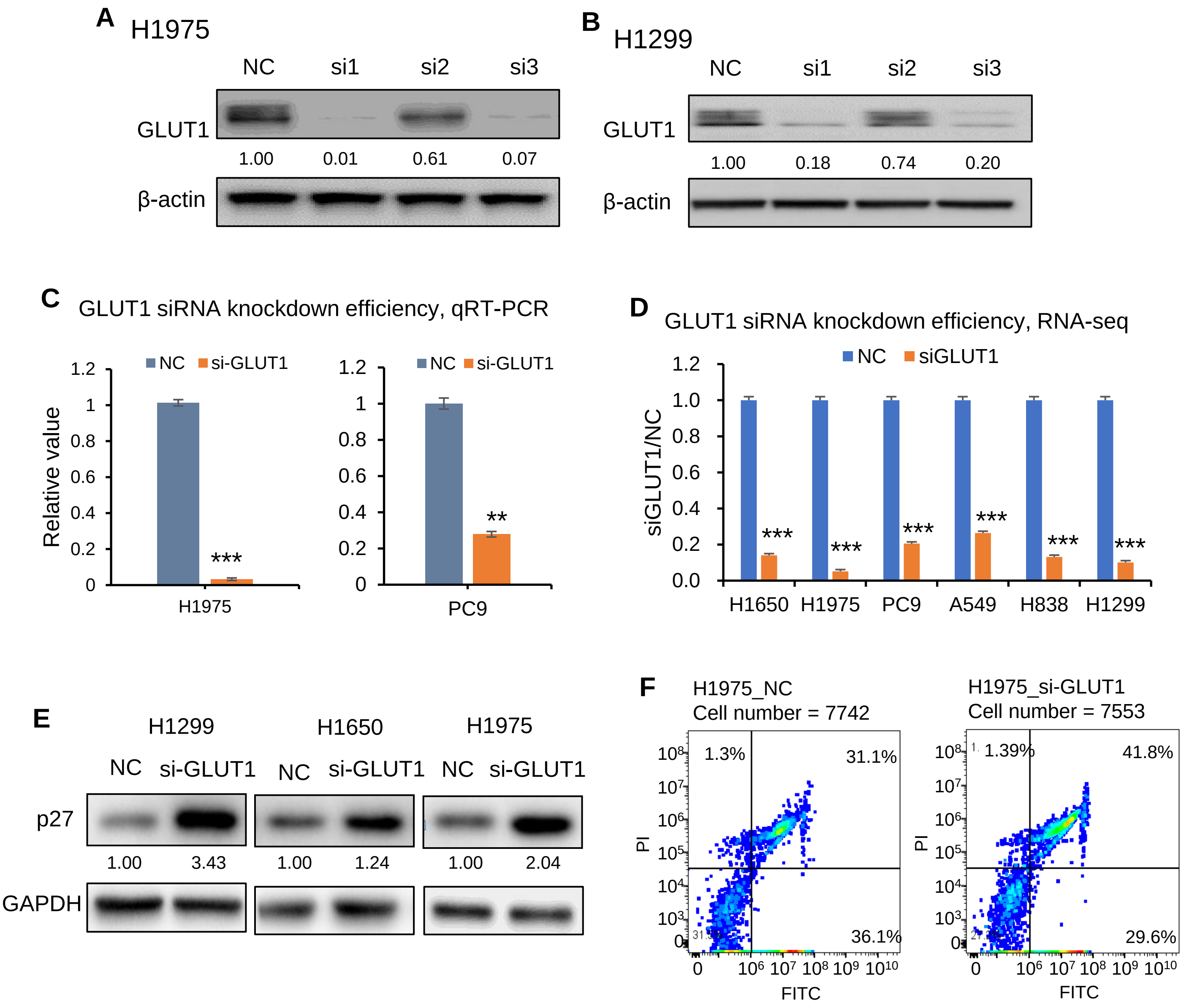

Figure S2. GLUT1 siRNA knockdown efficiency. **A**, **B**, GLUT1 protein expression was decreased upon siRNAs of GLUT1 transfected in lung cancer cells at 72 hours measured by Western blotting. Three different GLUT1 siRNAs sequences were tested; **C, D**, GLUT1 mRNA level was decreased upon siRNAs of GLUT1 transfected measured by qRT-PCR and RNA-seq. Mixed of 3 individual siRNAs was used; **E**, p27 protein was increased upon GLUT1 silencing; **F**, The percentages of apoptosis cells were increased after GLUT1 silencing in H1975 cell line.

Supplementary Figure S3

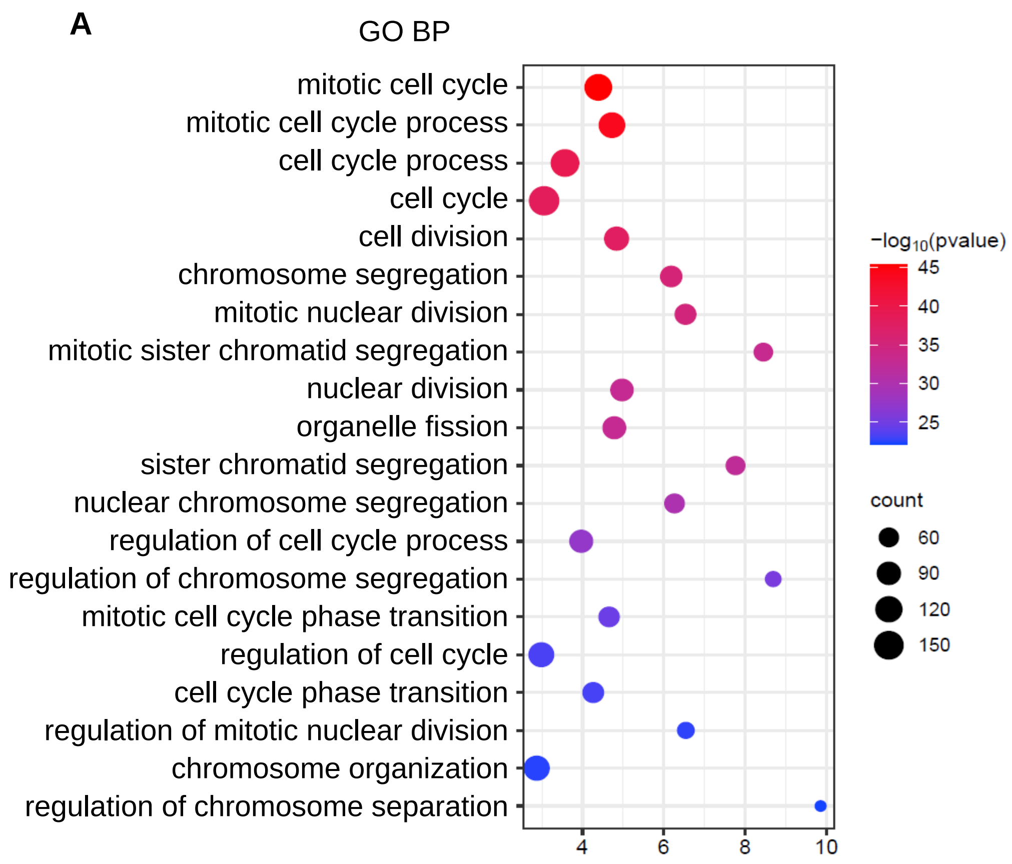

Figure S3. GLUT1 positive correlated genes involved in cell cycle in human LUAD tissues. A, There are 686 genes positively correlated with GLUT1 mRNA expression in LUAD tissues (Peason correlation, n = 464, average r > 0.34, p < 0.01). GO BP (Gene Ontology, Biology Process) analysis of these 686 genes in DAVID website showing that the cell cycle is on the top list..

Supplementary Figure S4

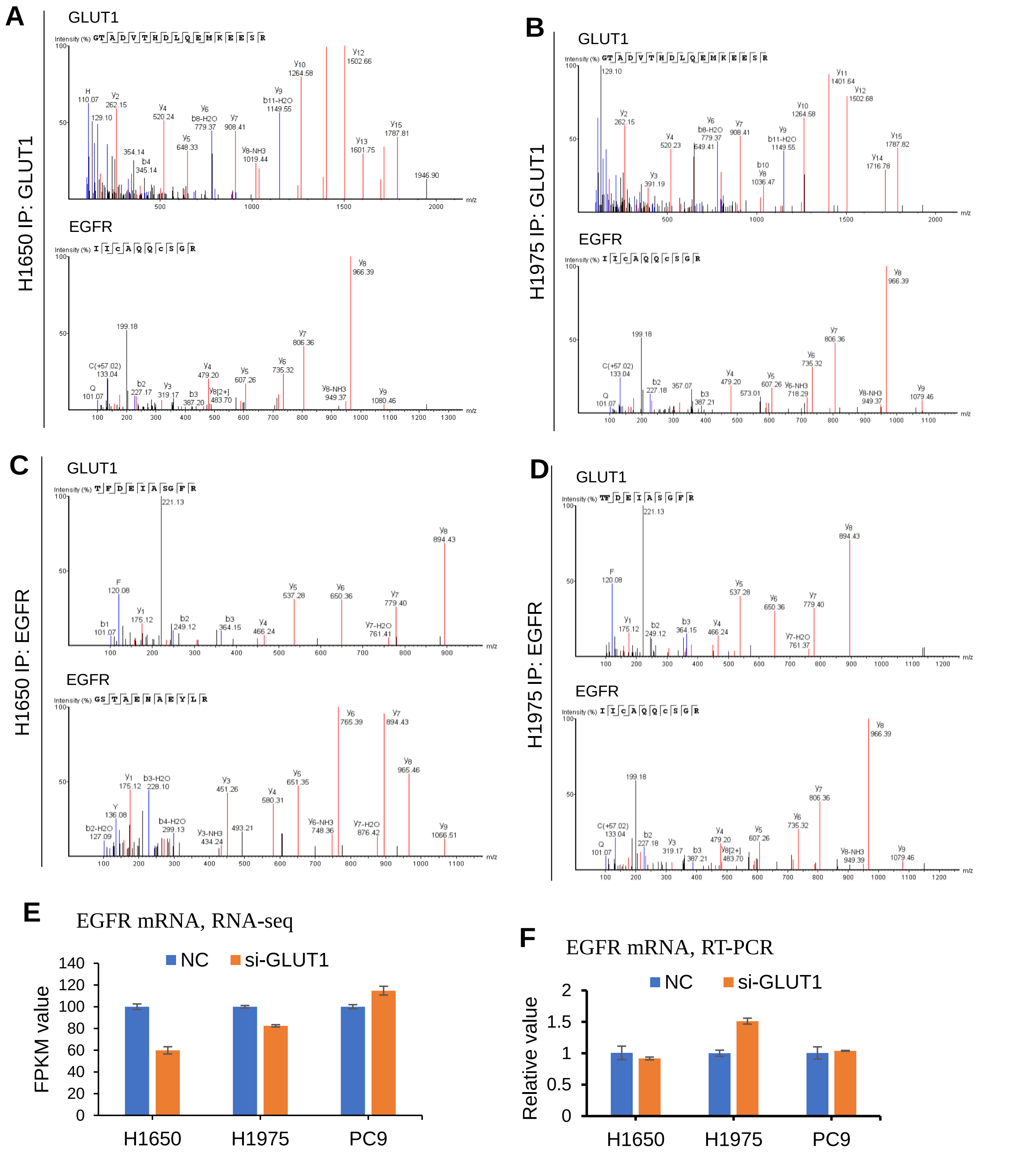

Supplementary Figure S4

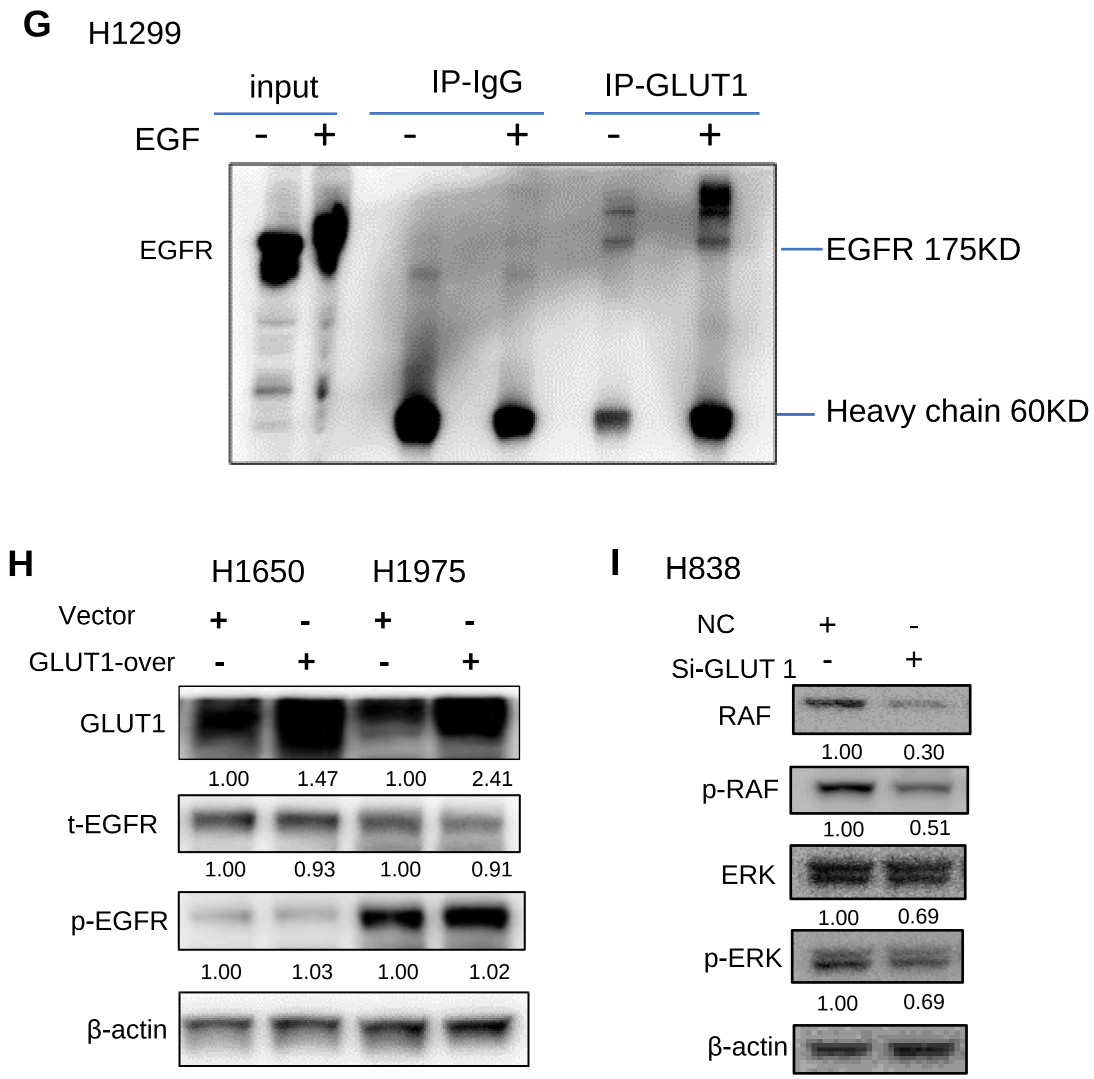

Figure S4. GLUT1 and EGFR have protein-protein interactions. A-D, The PSM (peptide spectrum match) of GLUT1 and EGFR in H1650 and H1975 cell lines. **E**, The EGFR mRNA expression upon GLUT1 knockdown measured by RNA-seq; **F**, The EGFR mRNA expression after GLUT1 silencing using RT-qPCR assay; **G**, A stronger EGFR binding band was detected upon EGF stimulated in H1299; **H**, Over-GLUT1 didn't increase EGFR protein level measured by Western blotting in H1650 and H1975 cell lines; **I**, RAF and p-ERK proteins were decreased after GLUT1 knockdown in H838 cells.

Supplementary Figure S5

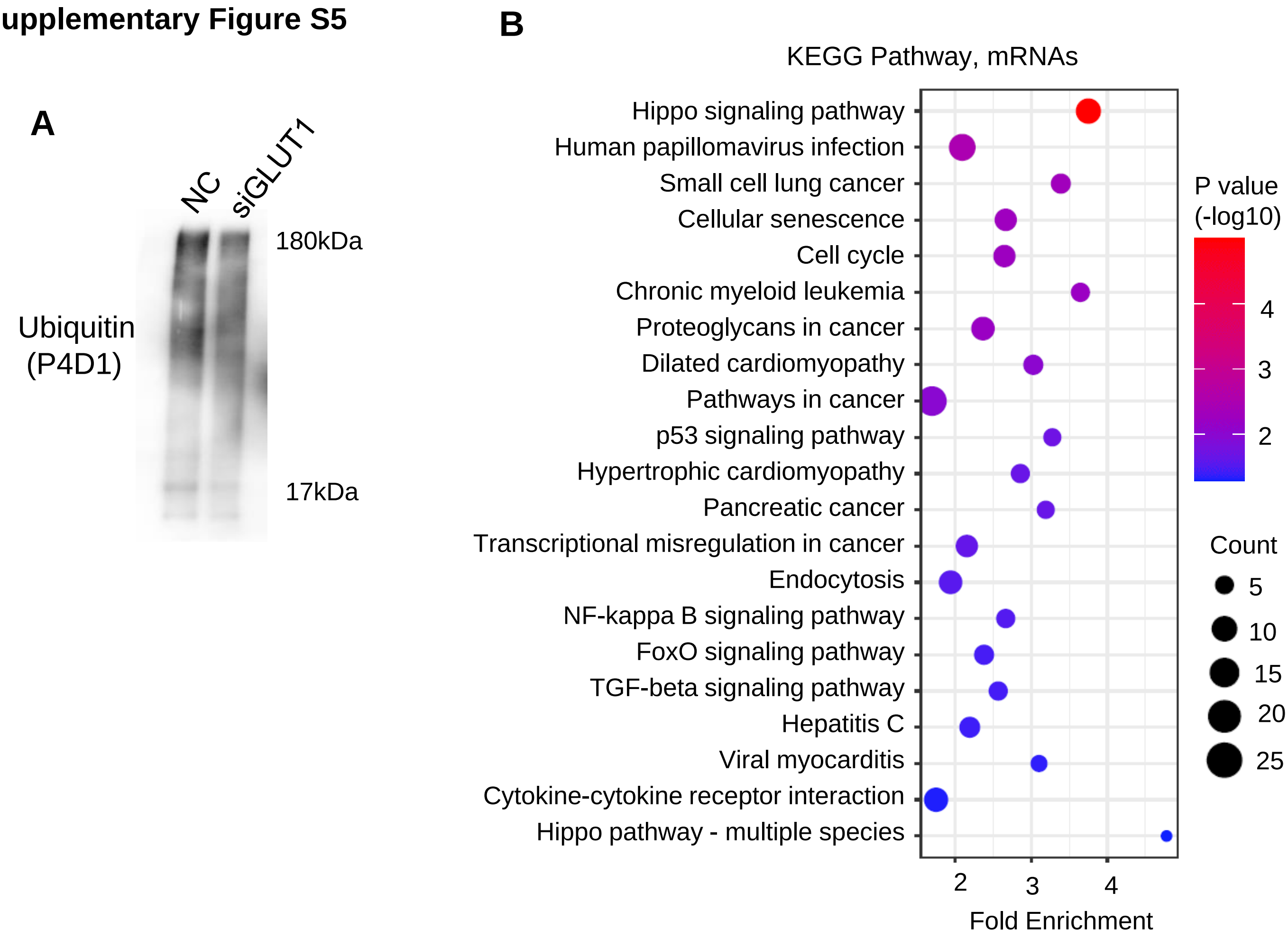

Figure S5. Several oncogenic signaling pathways are enriched upon GLUT1 silencing. A, The ubiquitination lever was decreased after GLUT1 knockdown using anti-ubiquitin antibody; **B**, KEGG pathway analysis of 616 down regulated genes (siGLUT1/NC control <0.65 in 2/3 cell lines) in RNA-seq data from three LUAD cell lines, PC9, H1650, and H1975. Hipo, Cell cycle, Pathways in cancer, p53,NF-kappa B, and TGF-beta signaling pathways were enriched. Note: Fold enrichment, the fold of rate that actual probability compares to random probability. Count, the gene number enriched in corresponding signaling pathways. P value, the confidence level of the enrichment result for each signaling pathway.

Supplementary Figure S6

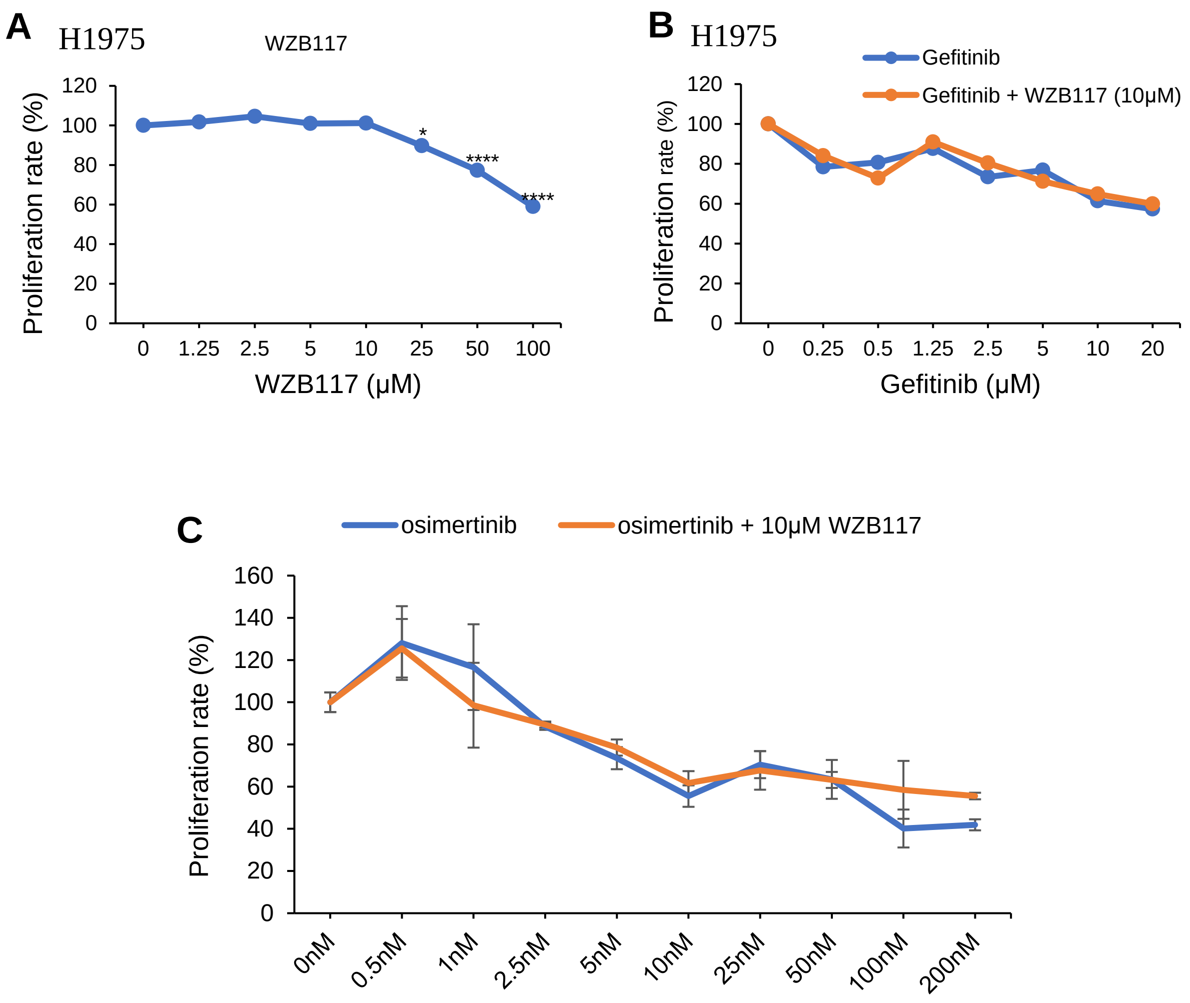

Figure S6. WZB117 doesn't enhance Gefitinib or Osimertinib sensitivity in EGFR T790M mutation cell line. A, Different dose of WZB117 treated in EGFR T790M mutation cell, H1975; **B**, Low concentration of WZB117 does not enhance Gefitinib sensitivity in H1975 cell line; **C**, Low concentration of WZB117 does not enhance Osimertinib sensitivity in H1975 cell line.

Table S1 siRNA sequence，PCR primer and regents

Reagent or Resource(5'-3')	Source	Identifier
**siRNA target sequence**		
UUCUCCGAACGUGUCACGUTT	GenePharma	Negative control siRNA target sequence-sense
ACGUGACACGUUCGGAGAATT	GenePharma	Negative control siRNA target sequence-antisense
GGAAUUCAAUGCUGAUGAUTT	GenePharma	GLUT1 siRNA target sequence-1-sense
AUCAUCAGCAUUGAAUUCCTT	GenePharma	GLUT1 siRNA target sequence-1-antisense
GCCCAUGUAUGUGGGUGAATT	GenePharma	GLUT1 siRNA target sequence-2-sense
UUCACCCACAUACAUGGGCTT	GenePharma	GLUT1 siRNA target sequence-2-antisense
GCAUCAACGCUGUCUUCUATT	GenePharma	GLUT1 siRNA target sequence-3-sense
UAGAAGACAGCGUUGAUGCTT	GenePharma	GLUT1 siRNA target sequence-3-antisense
**RT-PCR primers**		
CATGTACGTTGCTATCCAGGC	BGI Genomics	actin-F
CTCCTTAATGTCACGCACGAT	BGI Genomics	actin-R
AGGCACGAGTAACAAGCTCAC	BGI Genomics	EGFR-F
ATGAGGACATAACCAGCCACC	BGI Genomics	EGFR-R

Reagents

REAGENT or RESOURCE	SOURCE	IDENTIFIER
**Antibodies**		
Glut1 Rabbit mAb	Cell Signaling Technology	12939s
EGF Receptor (D38B1) XP® Rabbit mAb	Cell Signaling Technology	4267s
Phospho-EGF Receptor (Tyr1068) Antibody	Cell Signaling Technology	2234
Phospho-EGF Receptor (Tyr1086) Antibody	Cell Signaling Technology	2220s
Vimentin XP®Rabbit mAb	Cell Signaling Technology	5741s
Phospho-Vimentin Antibody	Cell Signaling Technology	13614s
E-Cadherin (24E10) Rabbit mAb	Cell Signaling Technology	3195s
N-Cadherin Antibody	Cell Signaling Technology	4061s
ZEB1 (D80D3) Rabbit mAb	Cell Signaling Technology	3396s
PARP	Cell Signaling Technology	9542s
Ubiquitin (P4D1) Mouse mAb	Cell Signaling Technology	3936s
β-Actin (13E5) Rabbit mAb (HRP Conjugate	Cell Signaling Technology	5125s
Anti-rabbit IgG	Cell Signaling Technology	7074s
Anti-mouse IgG	Cell Signaling Technology	7076s
**Chemicals, peptides, and recombinant proteins**		
PierceTM BCA Protein Assay kit	Thermo Fisher Scientific	23225
**Critical commercial assays**		
RPMI 1640	Gibco	C11875500BT
Fetal bovine serum	Gibco	10270-106
Penicillin-streptomycin	Gibco	15140-122
Phosphate Buffered Saline	Gibco	C10010500BT
Transwell of Falcone Permeable Supports	Falcon	C0121
Matrigel Basement Membrane Matrix	Corning	356234
RIPA buffer	Cell Signaling Technology	9806S
PMSF	Beyotime	ST506-2
Protease & phosphatase inhibitor	Thermo Fisher Scientific	1861280
RNA Lipofectamine RNAi max	Invitrogen	13778-150
Opti-MEM medium	Gibco	31985-062
Trypsin-EDTA (1X)	Gibco	25200-056
Methyl alcohol	Guangdong Guanghua	CAS67-56-1
Trizol reagent	Ambion	15596026
Tris-MOPS-SDS Running Buffer Powder	GenScript	M00138
SDS-PAGE gel	GenScript	M00654
TBS-T buffer (10x)	Boster Biological Technolc	AR0195-10
Prestained Protein Ladder	Gene Tech	R1001-002
CHX	MCE	HY-12320

MG-132	MCE	HY-13259
WZB117	MCE	HY-19331
Gefitinib	MCE	HY-50895
EGF	Peprotech	AF-100-15
crystal violet	Beyotime	C0121
Annexin V/PI kits	BD biosciences	AB_2869082
BSA	Sigma-Aldrich	9048468
UltraSignal ECL Reagent	4A Biotech	4AW011-500
Cell Counting Kit-8 (CCK-8)	Yeasen	40203ES92
Cell cycle and Apoptosis Analysis Kit	Beyotime	C1052
PrimeScript™ RT reagent Kit with gDNA Eraser (Perfect Real Time)	Takara	RR047A
TB Green Premix EX Taq II	Takara	RR820L
PVDF Western Blotting Membranes	Roche	3010040001
Tris-glycine-SDS transfer buffer (10x)	Boster Biological Technology	AR1152-10
**Deposited data**		
**Experimental models: Cell lines**		
H838	Cell Bank of the Chinese Academy of Sciences	CC0224
A549	Cell Bank of the Chinese Academy of Sciences	CC0202
H1975	Cell Bank of the Chinese Academy of Sciences	CC0206
PC9	Cell Bank of the Chinese Academy of Sciences	CC0204
H1299	Cell Bank of the Chinese Academy of Sciences	CC0203
H1650	Cell Bank of the Chinese Academy of Sciences	CC0211
**Software and algorithms**		
Image J	Image J	https://imagej.nih.gov/ij/download.html
GraphPad prism 7	GraphPad	https://www.graphpad.com/scientific-
R.4.2.0	R	https://www.r-project.org/
ClusterProfiler.4.4.4	ClusterProfiler	https://bioconductor.org/packages/release/bioc/html/clusterProfiler.html
hisat2.2.2.1	hisat2	https://daehwankimlab.github.io/hisat2/
FastQC.v0.11.9	FastQC	https://www.bioinformatics.babraham.ac.uk/projects/fastqc/
samtools.1.15	samtools	https://www.htslib.org/
stringtie.2.2.1	stringtie	https://ccb.jhu.edu/software/stringtie/

Table S2 Proteins binding to both EGFR and GLUT1 in H1975 and H1650 cells

Symbol	H1975 IgG -10lgP	H1975 IgG Area	H1975 GLUT1 -10lgP	H1975 GLUT1 Area	H1975 EGFR -10lgP	H1975 EGFR Area	H1975 GLUT1/IgG ratio	H1975 EGFR/IgG ratio	H1650 IgG-H1650 Gene_name_i	H1650 IgG -10lgP	H1650 IgG Area	H1650 GLUT1 -10lgP	H1650 GLUT1 Area	H1650 EGFR -10lgP	H1650 EGFR Area	H1650 GLUT1/IgG ratio	H1650 EGFR/IgG ratio
SLC2A1			190.03	7.47E+07	95.01	2.08E+06	#DIV/0!	#DIV/0!	SLC2A1			176.34	2.49E+07	56.16	6.50E+05	#DIV/0!	#DIV/0!
EGFR			107.31	1.41E+07	234.8	4.43E+07	#DIV/0!	#DIV/0!	EGFR			125.45	4.90E+06	212.86	2.36E+07	#DIV/0!	#DIV/0!
ACAT1	114.84	2.51E+06	199.56	4.00E+07	142.55	6.30E+06	15.9	2.51	ACAT1			109.55	4.28E+06	52.21	5.61E+05	#DIV/0!	#DIV/0!
ARL1			140.65	1.27E+07	25.19	1.82E+06	#DIV/0!	#DIV/0!	ARL1	58.7	4.54E+05	110.97	5.04E+06	98.28	3.63E+06	11.10	8.00
ASPH			180.17	2.22E+07	50.93	5.87E+05	#DIV/0!	#DIV/0!	ASPH			127.46	5.98E+06	54.56	5.77E+05	#DIV/0!	#DIV/0!
CCT6A	118.58	3.64E+06	203.92	4.93E+07	134.74	8.52E+06	13.5	2.34	CCT6A	99.39	1.36E+06	123.67	7.05E+06	112.67	3.36E+06	5.18	2.47
CFL1	91.16	3.02E+06	123.53	1.68E+07	138.12	1.13E+07	5.6	3.74	CFL1	97.01	2.49E+06	148.62	1.80E+07	88.5	5.08E+06	7.23	2.04
DARS1			201.66	3.12E+07	106.56	2.34E+06	#DIV/0!	#DIV/0!	DARS1			62.43	1.79E+06	126.06	2.81E+06	#DIV/0!	#DIV/0!
GALK1	29.68	3.11E+05	83.52	6.51E+06	61.49	1.23E+06	20.9	3.95	GALK1			21.53	7.19E+05	39.22	6.85E+05	#DIV/0!	#DIV/0!
GRN	105.86	1.47E+07	183.18	4.83E+07	138.63	3.21E+07	3.3	2.18	GRN			96.04	7.03E+06	61.17	2.26E+06	#DIV/0!	#DIV/0!
HADHA	49.95	2.19E+05	244.23	4.39E+07	75.5	5.86E+05	200.5	2.68	HADHA			274.04	8.68E+07	65.22	4.46E+05	#DIV/0!	#DIV/0!
KTN1			169.92	1.60E+07	47.49	1.36E+06	#DIV/0!	#DIV/0!	KTN1			37.86	5.14E+05	37.96	1.73E+05	#DIV/0!	#DIV/0!
MARS1	23.19	3.54E+05	208.6	3.56E+07	65.22	1.43E+06	100.6	4.04	MARS1	23.36	2.61E+05	69.87	2.47E+06	40.33	1.04E+06	9.46	3.98
NUMA1			105.68	4.19E+06	36.71	5.14E+06	#DIV/0!	#DIV/0!	NUMA1			91.64	8.40E+05	32.01	3.25E+05	#DIV/0!	#DIV/0!
NUP93			231.34	2.70E+07	65.01	5.72E+05	#DIV/0!	#DIV/0!	NUP93	79.71	1.20E+06	173.6	9.62E+06	144.73	3.24E+06	8.02	2.70
PARK7			96.78	3.76E+06	53.47	1.01E+06	#DIV/0!	#DIV/0!	PARK7			81.37	2.51E+06	53.81	1.37E+06	#DIV/0!	#DIV/0!
PGK1	85.9	2.53E+06	145.54	7.21E+06	168.56	1.21E+07	2.8	4.78	PGK1			26.05	5.15E+05	20.7	9.56E+05	#DIV/0!	#DIV/0!
PLS3			124.63	5.40E+06	28.72	1.77E+05	#DIV/0!	#DIV/0!	PLS3			49.74	4.85E+05	51.16	5.74E+05	#DIV/0!	#DIV/0!
PPP1CA			111.79	8.55E+06	47.62	7.11E+05	#DIV/0!	#DIV/0!	PPP1CA			123.35	1.09E+06	24.37	2.37E+05	#DIV/0!	#DIV/0!
PRDX1	140.04	5.06E+06	141.15	1.02E+07	168.94	1.85E+07	2.0	3.66	PRDX1	106.14	2.78E+06	171.37	3.60E+07	146.09	1.17E+07	12.95	4.21
PSME3			113.73	4.99E+06	69.43	1.81E+06	#DIV/0!	#DIV/0!	PSME3			39.38	9.39E+05	80.57	2.86E+06	#DIV/0!	#DIV/0!
PYCR1			115.18	7.77E+06	27.84	3.96E+05	#DIV/0!	#DIV/0!	PYCR1			77.3	7.42E+05	35.52	2.75E+05	#DIV/0!	#DIV/0!
SLC1A5			244.98	2.09E+08	51.17	3.48E+05	#DIV/0!	#DIV/0!	SLC1A5			219.64	1.03E+08	50.15	3.79E+05	#DIV/0!	#DIV/0!
TLN1	43.58	1.51E+05	252.35	3.82E+07	44.73	4.99E+05	253.0	3.30	TLN1	65.76	6.97E+05	142.27	4.49E+06	146.28	6.79E+06	6.44	9.74
UBA1	119.92	3.29E+06	221.13	2.57E+07	172.42	7.64E+06	7.8	2.32	UBA1			81.28	2.18E+06	76.71	5.06E+05	#DIV/0!	#DIV/0!
XRCC5			191.03	2.58E+07	72.77	6.47E+05	#DIV/0!	#DIV/0!	XRCC5			40.36	6.99E+05	31.64	2.34E+05	#DIV/0!	#DIV/0!
YBX3	168.07	2.54E+05	156.63	2.97E+06	139.8	8.71E+05	11.7	3.43	YBX3			149.24	2.20E+06	96.72	4.05E+05	#DIV/0!	#DIV/0!
